# Diabetes rescue, engagement, and management (D-REM) for hypoglycemia: Clinical trial protocol of a community paramedic program to improve diabetes management among adults with severe hypoglycemia

**DOI:** 10.1371/journal.pone.0322177

**Published:** 2025-06-09

**Authors:** Sumit Bhagra, Allison L. Ducharme-Smith, Michael B. Juntunen, Chad P. Liedl, Elizabeth H. Golembiewski, Wendy J. Sundt, Tami S. Krpata, Michelle A. Lampman, Anna L. Espinoza, Rozalina R. McCoy

**Affiliations:** 1 Endocrinology, Mayo Clinic Health System – Southeast Minnesota region, Austin, Minnesota, United States of America; 2 Division of Community Internal Medicine, Geriatrics, and Palliative Care, Mayo Clinic, Rochester, Minnesota, United States of America; 3 Mayo Clinic Ambulance Service, Mayo Clinic, Rochester, Minnesota, United States of America; 4 Division of Epidemiology, Mayo Clinic, Rochester, Minnesota, United States of America; 5 Research Services, Mayo Clinic, Rochester, Minnesota, United States of America; 6 Kern Center for the Science of Health Care Delivery, Mayo Clinic, Rochester, Minnesota, United States of America; 7 Division of Endocrinology, Mayo Clinic, Rochester, Minnesota, United States of America; 8 Division of Endocrinology, Diabetes, and Nutrition, University of Maryland School of Medicine, Baltimore, Maryland, United States of America; PLOS: Public Library of Science, UNITED KINGDOM OF GREAT BRITAIN AND NORTHERN IRELAND

## Abstract

**Background:**

Diabetes is among the most prevalent chronic conditions in the United States. Challenges in optimal diabetes care include fragmented care, gaps in diabetes self-management education, and high treatment burden. Severe hypoglycemia, a serious and potentially preventable event, indicates the need for treatment optimization. Inadequate or inaccessible care increases hypoglycemia risk. Community paramedics are well-positioned to fill these care gaps by providing focused diabetes self-management education and improving patient self-efficacy. Integrating community paramedics into care teams offers a novel pathway to improve diabetes outcomes.

**Methods and analysis:**

We will conduct a pragmatic 2-group, parallel-arm, randomized clinical trial of a community paramedic–led “Diabetes Rescue, Engagement, and Management” program to enhance diabetes self-management in patients with a history of hypoglycemia. The study will enroll 150 adults (≥18 years) with diabetes and a history of level 3 hypoglycemia from 5 counties in Minnesota. Participants identified as having hypoglycemia (from an integrated health system and the primary ambulance service in the area) will be randomly assigned to the program intervention or to usual care. The intervention group will receive community paramedic home visits for approximately 1 month to deliver diabetes self-management education tailored to individual needs. Both groups will receive written diabetes education and resource materials. Outcomes include change in diabetes self-management, hypoglycemia, hyperglycemia, hemoglobin A_1c_ level, diabetes distress, and health-related quality of life, assessed at baseline, 1 month, and 4 months. Qualitative interviews of 16 intervention participants and 16 persons who decline participation will be analyzed to understand the program’s effects and reasons for nonparticipation, to inform future program design.

**Trial registration:**

ClinicalTrials.gov NCT04874532

## Background and rationale

Diabetes ranks among the most prevalent and costly chronic diseases in the US, affecting more than 38.4 million people, or 11.6% of the US population [1]. The goal of glucose-lowering treatment is to prevent both acute and chronic diabetes complications [2–7]. Preventing hypoglycemia, particularly severe hypoglycemia, is a key component of diabetes management because hypoglycemia is associated with cardiovascular events [8–12], mortality [8,10–18], decreased quality of life [19], disability [20], cognitive impairment [21–23], and high health care costs [20] and is preventable with optimal care. Despite the continued focus on hypoglycemia prevention, rates of severe hypoglycemia among people living with diabetes [24,25], as well as mortality after hypoglycemic events [26], remain high. These outcomes are influenced, in part, by the difficulty in preventing severe hypoglycemic events among those at risk.

Prior hypoglycemia is one of the strongest risk factors for future hypoglycemia [27–33]. Thus, we need comprehensive, accessible, and sustainable interventions to improve diabetes self-management and prevent hypoglycemia among patients with diabetes. The interventions published thus far in pursuit of these goals range from hypoglycemia prevention education imparted by a pharmacist [34] to a structured nurse-led outreach program after an acute episode of hypoglycemia requiring emergency care [35]. Most of these programs are broad in scope; evaluate surrogate outcomes such as rates of glucagon prescription, improved recognition of hypoglycemia [34], or improved engagement in self-management [36]; and may be challenging to implement in resource-limited settings.

The need is particularly urgent for interventions that can be scaled to less-resourced settings and populations, including rural communities where access to specialty diabetes care and diabetes self-management education and support (DSMES) is lacking [37–39]. Community paramedicine—in which paramedics with additional training in nonemergency medicine, social determinants of health, and chronic disease management deliver services outside the typical emergency response model—has gained traction across the US as a scalable, impactful, and efficient approach to health care delivery in underserved communities and populations [12,40–46]. Unlike traditional emergency response services, community paramedics (CPs) prioritize preventive care with a strong focus on primary care delivery, education, prevention, and overall wellness [12,45–49]. Two core frameworks of community paramedicine make it ideally positioned to meet the multifaceted demands of rural and underserved communities [50–52]. First, the primary health care framework of community paramedicine focuses on preventing hospital or emergency department (ED) admissions/readmissions and monitoring chronic illness [7,42,46,53]. Second, the community coordination framework seeks to connect patients to appropriate social, community, and medical services [45,46]. Both frameworks are optimally suited to addressing the complex and multifaceted health needs of people with diabetes experiencing severe hypoglycemia.

Historically, community paramedicine care models have focused on patients with a history of frequent hospital, ED, and/or emergency medical services (EMS) utilization in the setting of multimorbidity and frailty [7,42,46,53–57], with the goal of reducing their acute health care utilization. However, CPs in many states have an increasingly broad scope of practice that could allow them to deliver DSMES with oversight by a physician medical director. We are aware of only 1 study that evaluated the feasibility of a CP-led intervention after an acute episode of hypoglycemia requiring EMS activation [58]. That study noted a significant increase in participants’ self-efficacy in managing hypoglycemia but also an attrition rate of nearly 70% during the 8-week study period, which was attributed to the high prevalence of low literacy and adverse socioeconomic factors in that geographical region [58].

Given this knowledge gap, we established a CP-led DSMES program for adults with diabetes—“Diabetes Rescue, Engagement, and Management” (D-REM). This program seeks to both decrease acute care utilization and improve self-management, glycemic management, and quality of life. We recently completed a prospective single-arm study focused on adults with elevated hemoglobin A_1c_ (HbA_1c_) levels who had an ED visit or hospitalization for any reason within the previous 6 months (NCT04385758) [59] to evaluate the feasibility, acceptability, and effectiveness of D-REM in this patient population; results will be forthcoming in a separate publication. We now seek to examine the feasibility, effectiveness, and acceptability of D-REM for patients who had experienced level 3 hypoglycemia in a prospective, parallel-arm, pragmatic randomized clinical trial (RCT) (NCT04874532).

## Methods and analysis

This study has been approved by the Mayo Clinic Institutional Review Board (IRB; #20–006799) and registered on ClinicalTrials.gov (NCT04874532). The protocol description and consent form is provided in the S1 Appendix. Protocol modifications will be reviewed by the IRB and communicated to trial participants, the research team, and ClinicalTrials.gov as appropriate.

### Study aims

#### Aim 1.

The first aim is to evaluate the effectiveness of the program “D-REM Hypoglycemia” through a pilot, 2-group, parallel-arm, pragmatic RCT carried out in the community setting in 5 counties of southeast Minnesota (Freeborn, Mower, Olmsted, Steele, and Wabasha). The intervention group will receive CP home visits and telephone calls for approximately 1 month (tailored to the patient’s clinical situation and need). Both the intervention and usual care groups will be provided with printed diabetes education materials and a resource guide for accessing diabetes-related care in addition to usual care. Aim 1 will assess program feasibility and efficacy, with the primary outcome of diabetes-self management, measured by the Diabetes Self-Management Questionnaire (DSMQ) [60]. Secondary outcomes will include recurrent hypoglycemia, severe hyperglycemia, HbA_1c_ levels, diabetes distress, and health-related quality of life.

#### Aim 2.

The second aim is to identify features of D-REM Hypoglycemia that are most meaningful to enrolled participants through participant interviews. This will allow us to better understand whether and how specific program components (e.g., home visit, environmental/behavioral assessment, active learning) addressed participants’ needs related to managing their diabetes.

#### Aim 3.

Participants eligible for Aim 1 who choose not to participate in the D-REM Hypoglycemia study will be invited to participate in a 1-time semistructured qualitative interview to understand the reasons for program decline, the types of programs and/or interventions that people with diabetes who had experienced level 3 hypoglycemia believe to be beneficial and desired for them, and their overall experience with hypoglycemia and diabetes management. This will allow us to better tailor interventions to support people with diabetes experiencing level 3 hypoglycemia and help elucidate the factors contributing to lack of participation in community paramedicine programs [58].

### Study design

**Fig 1:**
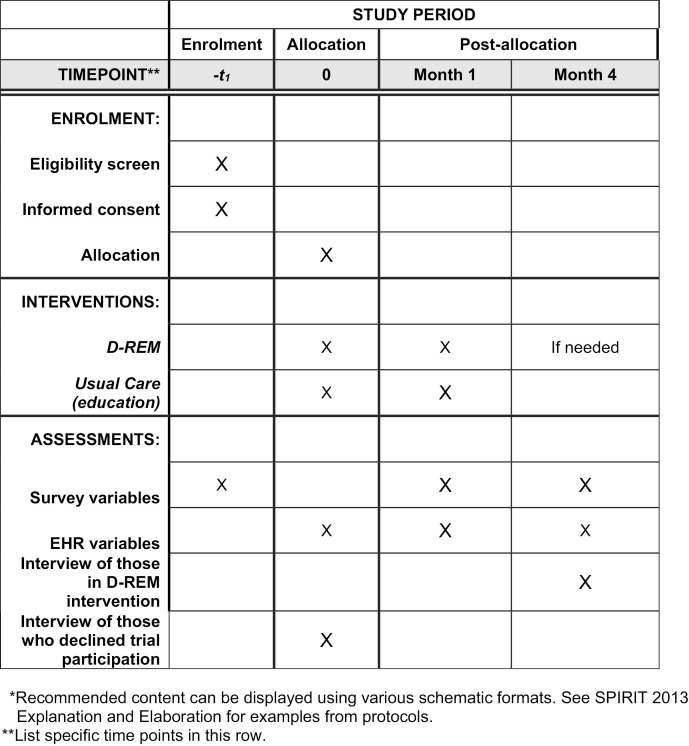
Example template of recommended content for the schedule of enrolment, interventions, and assessments.* D-REM indicates Diabetes-Rescue, Engagement, and Management Intervention; EHR, electronic health record.

The study design is a prospective, pragmatic, 2-group, parallel-arm RCT. The study and enrollment began on June 4, 2021, with an anticipated enrollment completion date of July 2025. We anticipate data collection to be completed by December 31, 2025, and results are expected by December 21, 2026. No stage of the study is currently complete. Fig 1 shows the schedule of enrollment, interventions, and assessments.

### Setting

Mayo Clinic operates an integrated health care delivery system, caring for patients locally, regionally, nationally, and internationally, with a primary hub located in Rochester, Minnesota (Olmsted County). The primary care practices at Mayo Clinic in Rochester—encompassing internal medicine, geriatrics, family medicine, and pediatrics—provide services to Mayo Clinic employees, their dependents, and local residents of Rochester and the surrounding area. Mayo Clinic Health System (MCHS) functions as a network of community-based clinics, hospitals, and health care facilities in Minnesota and Wisconsin, offering both primary and specialty care to local communities. Mayo Clinic and MCHS share a single electronic health record (EHR) system, but their primary care coverage areas do not overlap.

Mayo Clinic Ambulance holds accreditation from the Commission on Accreditation of Ambulance Services (ground ambulance) and the Commission on Accreditation of Medical Transport Systems (ground and air ambulance), and is an Accredited Center of Excellence (emergency communications center). It operates as the main advanced life support provider across 14 locations in eastern and central Minnesota and western Wisconsin, encompassing 6,894 square miles of urban, suburban, and rural areas. The service is staffed by emergency medical technicians, paramedics, and registered nurses and responds to approximately 100,000 service requests annually. The Mayo Clinic Ambulance Community Paramedic Service Minnesota team is based in Olmsted County and serves the southeast Minnesota region of Olmsted, Freeborn, Mower, Steele, and Wabasha counties. CPs care for patients in the home during prescheduled nonemergent appointments.

### Study participants

#### Aim 1.

Eligible participants will be persons aged 18 years or older with type 1 or type 2 diabetes, who are able to provide informed consent, had experienced level 3 hypoglycemia within 6 months of outreach, and live in Freeborn, Mower, Olmsted, Steele, or Wabasha counties in Minnesota. These areas have high prevalence of diabetes, few diabetes providers, and an established Mayo Clinic Ambulance CP service infrastructure. Patients must be empaneled to a primary care clinician at Mayo Clinic in Rochester or at MCHS to enable CPs to deliver DSMES and coordinate care with the patient’s primary care clinician and other diabetes care team members. The total number of patients to be included will be 150 participants for Aim 1 (75 participants in each arm).

Potential participants experiencing level 3 hypoglycemia will be identified by using 3 complementary approaches and data sources, although each potential participant will be offered the intervention only once. The 3 complementary approaches were selected because the majority of severe hypoglycemic events are treated by caregivers at home, with some culminating in an EMS call. Approximately half of hypoglycemic events managed by EMS are treated on scene without transport to the ED or hospital [61–64]. Hypoglycemic events among nontransported patients are likely to be missed by the health care team, with no intervention provided, especially because patients typically do not report these events to clinicians [65–67] and clinicians do not routinely screen at-risk patients for hypoglycemia [68].

**Source 1: Patients who are treated for hypoglycemia by Mayo Clinic Ambulance.** The Mayo Clinic Ambulance EHR system, emsCharts Tablet (or emsCharts NOW; Zoll Data Systems), will be used to generate a report of events for hypoglycemia, identified using the discrete fields of glucose value on scene (<54 mg/dL to identify level 2 hypoglycemia) and medication administration (glucose; dextrose 10%, 25%, or 50% in water; or glucagon). A real-time report will be generated by FirstWatch (a practice/quality tool used by Mayo Clinic Ambulance) based on these criteria and emailed to the study team. The report contains information about the ambulance encounter, including patient self-reported name, self-reported date of birth, encounter address, event narrative, encounter diagnostics (including glucose values), and encounter treatments. This information will be used by the study team to identify the patient’s Mayo Clinic medical record number, if one exists, and screen them for eligibility for the study.

**Source 2: Patients who were treated in the Mayo Clinic or MCHS ED or hospital for hypoglycemia.** We will use the EHR to identify patients with an *International Classification of Diseases, Tenth Revision, Clinical Modification* code for hypoglycemia present on an ED claim or on a hospital claim on the day of either hospital admission or discharge. These charts will be reviewed approximately monthly to identify patients meeting eligibility criteria, removing duplicates of previously identified patients.

**Source 3: Patients who self-report experiencing level 3 hypoglycemia**. These patients will be identified through 2 methods. 1) Recruitment letters will be sent via the patient portal or postal mail to patients at risk for hypoglycemia, informing them about the study and inviting them to enroll if they have experienced level 3 hypoglycemia. 2) Study information will be disseminated to primary care clinicians, endocrinologists, certified diabetes care and education specialists, medication therapy management pharmacists, dieticians, and primary care team nurses (who assist clinicians with diabetes management) to make them aware of the D-REM program and encourage them to make referrals as clinically appropriate.

A study team member will review the EHR to confirm eligibility of all identified patients and further exclude patients based on the following criteria: 1) cognitive impairment that prevents informed consent; 2) insufficient conversational English skills; 3) residence in a long-term care facility; 4) enrollment in hospice; 5) participation in another care coordination or disease management program; or 6) presence of advanced or terminal illness.

#### Aim 2.

Aim 2 is a qualitative study involving a subset of participants who had been randomly assigned in Aim 1 to the intervention arm and completed the D-REM program. We will use stratified purposeful sampling to intentionally select approximately 16 participants to represent differing demographic, clinical, and geographic subgroups [69–73].

#### Aim 3.

Participants who are eligible for Aim 1 but decline participation in the D-REM study will be offered enrollment into Aim 3 during the same initial outreach telephone call.

### Informed consent procedures

#### Aim 1.

Written consent will be obtained via mailed or electronic forms, depending on participant preference. Both options will be available to reduce barriers to participation by rural, older, and low-income individuals who may have limited broadband internet access (requiring paper forms) and to increase completion rates (making electronic forms available). At the same time, participants will be sent a baseline survey (paper or electronic versions). No remuneration will be provided, although the D-REM program will be offered at no cost to the patient. The consent will include a provision that deidentified study data may be used for future research or shared with other researchers without additional informed consent after approval of an affiliated IRB.

#### Aim 2.

Consent for the interview is included in consent procedures for Aim 1. No new consent will be obtained. No remuneration will be provided.

#### Aim 3.

Oral consent will be obtained for participation in this part of the study. Participants will receive $25 in remuneration for completing the interview.

### Aim 1 description

#### Randomization.

After receipt of the signed consent form and baseline survey, participants will be randomly assigned by the study staff, 1:1, to intervention vs control arms, with blocking by diabetes type (1 vs 2) and how the hypoglycemic event was managed (caregivers or bystander without calling for medical attention vs EMS only vs ED/hospital). REDCap (Research Electronic Data Capture) database software will be used for randomization and allocation concealment. The system does not release the randomization code until the participant is enrolled. Participants will be sequentially recruited until the target accrual of 150 participants (75 per arm) is reached. Blinding is infeasible in this study.

#### Usual care.

Upon enrollment, study staff will provide each participant in both the usual care and intervention arms with a diabetes resource card. This card will include a list of clinic and community-based diabetes resources. Clinic resources will provide details on various care team members, such as primary care clinicians, nurses, pharmacists, social workers, diabetes care and education specialists, endocrinologists, and registered dietitian nutritionists, as well as instructions on how to schedule appointments with them. Community resources include community health workers, registered dietitian nutritionists (available in local grocery stores), family services, food banks, and regional wellness programing [74]. Participants will also receive a diabetes education packet containing information on diabetes self-management, nutrition, physical activity, glucose self-monitoring, and managing hypoglycemia. These materials will ensure that all participants receive the current standard of care after experiencing severe hypoglycemia. There are no restrictions on concomitant care and interventions during the trial.

#### Intervention.

After the consent form is returned, participant contact information will be communicated to the CP team, who will arrange further contact as part of the intervention.

**Fig 2:**
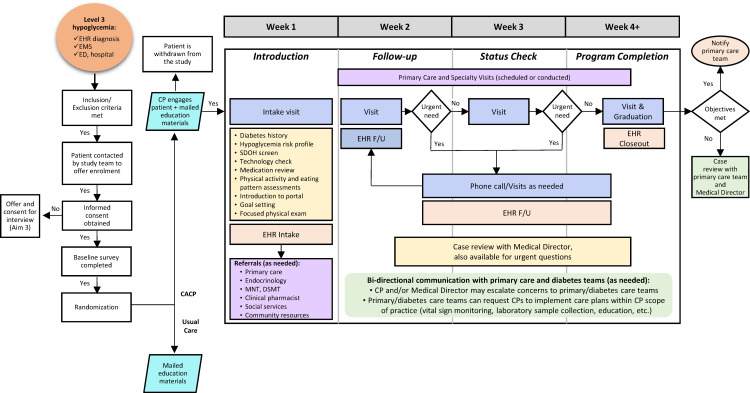
Schematic of the Diabetes-Rescue, Engagement, and Management Intervention. CP indicates community paramedic; DSMT, diabetes self-management training; ED, emergency department; EHR, electronic health record; EMS, emergency medical services; F/U, follow-up; MNT, medical nutrition therapy; SDOH, social determinants of health.

An overview of the D-REM clinical program is shown in Fig 2. The CP will meet patients at their home (preferred) or other mutually agreeable patient-requested location. Patients will undergo an in-person interview and examination lasting approximately 1 hour, with follow-up scheduled according to individual needs. On average, we anticipate each patient will have 2 1-hour in-person visits and 2 30-minute telephone visits. However, CPs can provide more frequent visits as clinically necessary for the patient.

During each visit, CPs will conduct assessments of health status and social determinants of health, perform a physical examination, review medications and provide counseling, as well as establish and review patient care goals. Guided by the participant’s needs, CPs can engage clinical resources (e.g., primary care, endocrinology, wound care, pharmacy, social services), activate community resources (e.g., meal delivery, food bank, social support), deliver hands-on education (e.g., help with meal preparation to teach about healthy eating patterns), or carry out clinician orders as part of usual care (e.g., vital sign monitoring, obtaining urine/blood samples). Bidirectional communication with the primary care team and the patient’s diabetes care team will occur via the EHR. CPs will be supported by their medical director and study principal investigator, who is an endocrinologist and general internist, and an endocrinology registered nurse/certified diabetes care and education specialist. Participants will be able to terminate their participation in the program at any time. The CP service will follow internal procedures for termination of care for reasons of staff or patient risk.

Upon completion of the program, participants will be transitioned to their primary care team. CPs will communicate directly with the primary care team to ensure continuity of care.

### Study procedures

#### Aim 1.

Participant data will be collected at baseline (before the first CP visit), 1 month after completing the first CP visit (to assess immediate program effect), and 4 months after completing the first CP visit (to assess short-term durability of program effect).

The EHR will be used to determine patient age, gender, race/ethnicity, urban vs rural status of primary residence location, medication use, comorbid conditions, and previous EMS/ED/hospital utilization related to hypoglycemia and hyperglycemia. Diabetes type and duration will be ascertained with the baseline survey. Primary and secondary end points will be measured via surveys conducted at baseline, 1 month, and 4 months. All surveys will either be mailed via US Postal Service or sent electronically by the Mayo Clinic Survey Research Center or administered in real time, over the telephone, with the study coordinator. If a participant is not reached after 2 call attempts, for each survey time point, second mailings will go out to nonresponders after 3 weeks. If participants do not respond to the second mailing, they will be contacted by a study team member by telephone, one last time, to remind them to complete the surveys. Additional copies will be mailed if necessary.

Surveys will assess the primary outcome of diabetes-self management (with the DSMQ [60]), which has been associated with glycemic management [75] and microvascular complications [76]. Secondary outcomes, also ascertained via survey, include self-reported hypoglycemia (glucose <54 mg/dL or need for third-party assistance), hyperglycemia (glucose ≥250 mg/dL), diabetes distress (with the Diabetes Distress Scale [DDS] [77,78]), and quality of life (with the 5-level EQ-5D version [EQ-5D-5L] [79,80]). The EHR will be used to identify EMS/ED/hospital utilization for hypoglycemia and hyperglycemia during the study period.

This is a minimal risk study, and no adverse effects of an educational intervention are anticipated. Nevertheless, participants will be provided with contact information for the Mayo Clinic IRB, the study principal investigator, and a study staff member with guidance on how to contact them with any concerns that may arise. All reported concerns will be treated as adverse events and reported to the IRB.

#### Aim 2.

A 1-time interview will be conducted by telephone or videoconferencing technology, recorded, and professionally transcribed.

#### Aim 3.

A 1-time interview will be conducted by telephone or videoconferencing technology, recorded, and professionally transcribed.

### Data management and safety

EHR data will be abstracted electronically by approved research staff. Self-administered surveys will be made available in both electronic and paper forms to reduce barriers to completion. Surveys will be distributed, collected, recorded, and stored by the Mayo Clinic Survey Research Center. The study principal investigator and statisticians will have access to the final trial data set, with additional access provided to other approved members of the research team as necessary for approved research activities.

All records linking study identification numbers to participant names will be securely stored and deleted after the project is completed. Research data will be accessible only with password-protected and logged access in a secure server. In accordance with standard guidelines, 5 years after the publication of the results, all paper copies of the forms and the analytical database will be destroyed.

The privacy of all participants will be protected by use of unique study IDs, with no use of participant names in any research data. The link between the study ID and the participant’s name will be stored in REDCap, a password-protected system available only to approved study staff. The potential risks of loss of confidentiality will be minimized by ensuring that data are stored only in designated locations, all study-related communication occurs in secure platforms, and data are accessed only by research team members and for specific study-related purposes.

### Outcomes

#### Primary outcome.

Analysis of covariance (ANCOVA) will be used to analyze the mean difference in the DSMQ score between arms at 1 month, adjusting for baseline scores. The DSMQ was chosen because its 5 subscales capture the wide range of factors that impede optimal diabetes care and may increase the risk of hypoglycemia, including dietary control, medication adherence, blood glucose monitoring, physical activity, and physician contact. To assess changes from baseline at 1 month and 4 months, we will use repeated-measures analysis of variance (ANOVA) via a modified mixed model. This method produces the least biased results when conducting an intention-to-treat analysis and allows for missing data, unlike standard ANCOVA. We will compare baseline demographics between arms by using *t* tests or the Kruskal-Wallis H test for continuous variables and the χ^2^ test or Fisher exact test for categorical variables; characteristics imbalanced between arms at a *P* < .05 level will be included as covariates in the final model.

#### Secondary outcomes.

Binary outcomes (self-report of hypoglycemia and hyperglycemia) will be analyzed using logistic regression to generate odds ratios with 95% CIs for comparison between arms. Normally distributed continuous outcomes (DDS, EQ-5D-5L) will be examined for differences at the 1-month mark with paired *t* tests. Because we anticipate some sum and subscale scores to be nonparametric, those differences in scale scores between arms at 1 month will be analyzed with ANCOVA, adjusting for baseline scores. As with our primary outcome, overall change from baseline at 1 month and 4 months will be assessed with repeated-measures ANOVA via a modified mixed model. Models will be adjusted for baseline characteristics as described above.

Aim 2 of the study is a qualitative assessment of participants’ perceptions of D-REM and its impact on their diabetes management. Our objective is to gather a rich description of experiences and insights related to the D-REM intervention from study participants. As such, sampling is guided by the principle of qualitative clarity as an analogue to the quantitative concept of statistical power [81]. We will use stratified purposeful sampling to intentionally select participants to represent predefined and explicit traits or conditions [81,82]. This will ensure a rich description across patients of different demographic characteristics and clinical profiles. Our a priori objective is to capture the perspectives of participants with type 1 and type 2 diabetes; younger and older age; those referred from the outpatient setting, transported to the ED, and treated by EMS without transport; and those living in a rural area and in a more urban setting. We anticipate that approximately 16 participants would allow us to sample a representative population, but the ultimate sample size will depend on the number needed to inform the key elements of the phenomenon studied [83]. Thus, when no new concepts or themes emerge from the interviews, data saturation will be reached. To determine the point of saturation, data analysis will be conducted concurrently and iteratively with data collection [83]. Participants will be recruited until information redundancy (thematic saturation) in their interview responses is reached [84,85].

Aim 3 of the study is a qualitative assessment of participants’ perceptions of hypoglycemia and services/programs to reduce hypoglycemia risk. We expect to reach thematic saturation with 16 participants. To determine when data saturation occurs, analysis will occur concurrently and iteratively with data collection [83]. Participants will be recruited until information redundancy (thematic saturation) in their interview responses is reached [84,85].

### Data analysis

#### Aim 1.

Analysis for all participants will occur in the arm in which they were assigned, in accordance with the intention-to-treat approach. Descriptive statistics will be presented as mean (SD) for continuous variables and frequency (percentage) for categorical variables. We will assess any potential imbalances in baseline characteristics between arms with the *t* test or the Kruskal-Wallis H test for continuous variables and χ^2^ or Fisher exact test for categorical variables. The primary outcome of this aim, change in DSMQ score from baseline to 1 month, will be analyzed by using ANCOVA, adjusting for baseline patient scores. Overall change in DSMQ from baseline to 4 months will be assessed by using repeated-measures ANOVA via a modified mixed model. Secondary outcomes will be analyzed for differences at 1 month by using paired *t* tests for normally distributed data and ANCOVA for nonparametric data. For binary outcomes, logistic regression will be performed, adjusting for baseline characteristics. When necessary and appropriate, multiple imputation will be used for missing values. No interim analyses are planned. An exploratory analysis will be conducted by diabetes type.

#### Aim 2.

In this qualitative aim we will use an in-depth interview method with semistructured interview guides to explore and understand participant perceptions of D-REM and its impact on diabetes management. These perspectives will also help us identify areas for program improvement. An experienced qualitative interviewer will conduct the interviews by telephone, with each session lasting between 45 and 60 minutes. All interviews will be audio recorded and transcribed verbatim by a trained transcriptionist.

A thematic approach will be employed for the qualitative assessment of individual interviews [86,87]. Preliminary data analysis will occur alongside data collection to identify the point of saturation [71–73]. Two study members will review each transcript multiple times, noting their initial impressions and providing descriptions of the data through analytic memos. These notes will then be used to create labels or codes, which will be arranged into higher-order categories to represent key themes related to the study aims. To facilitate data queries for analysis, a coding framework will be applied to transcripts by at least 2 trained coders who will discuss and come to agreement before entering data into NVivo 10 software (Lumivero). Data interpretation will include situating findings within existing knowledge or theory related to diabetes self-management and risks of hypoglycemia. The involvement of multiple analysts and review of findings by a multidisciplinary team will be used as a check against interpretive bias [81,82,84,88,89]. An analysis audit trail will document decisions made during the analyses.

#### Aim 3.

In this qualitative aim we will use an in-depth interview method with semistructured interview guides to explore and understand participant perceptions of hypoglycemia and programs/services that may reduce the risk of hypoglycemia. These perspectives will also help us identify areas for program improvement.

The purpose of this qualitative aim is to explore and understand patient perceptions of severe hypoglycemia risk factors, experiences, and precipitating factors; understand which services or resources can help prevent hypoglycemia; and understand why the interviewees were not interested in the D-REM intervention. Semistructured interview guides will include open-ended questions in the following domains: 1) participants’ perception of the patient’s severe hypoglycemia risk; 2) what they believe caused their prior hypoglycemic events or predisposed them to experiencing hypoglycemia; 3) how serious they believe these events to be; 4) which interventions, treatment regimen changes, and/or support systems could have prevented their earlier events and may help prevent future events; 5) what were the reasons for them declining participation in D-REM; and 6) what could be improved or changed about the D-REM program if it were to be incorporated into clinical practice. The purpose of the patient questions is to understand whether patients view themselves as at risk, which factors inform their assessments of risk, and what can be done to lower their risk. These perspectives will help us determine which patient-reported factors should be considered in designing interventions to reduce the risks of hypoglycemia for patients with diabetes.

An experienced interviewer will conduct the interviews, each lasting 45–60 minutes by telephone or videoconference. All interviews will be audio recorded and transcribed verbatim by a trained transcriptionist.

Qualitative assessment of individual interviews will use a conventional or inductive content analysis approach [85], which is appropriate when the existing literature is limited. Data analysis will be concurrent with data collection. Qualitative analysis will begin with 2 study team members reviewing each transcript several times and making notes of their initial impressions and descriptions of the data (analytic memos). Transcripts will be imported into Nvivo 10 software. Two coders will begin coding data line-by-line to generate a codebook. Coders will meet weekly to discuss emerging codes. The codebook will be refined during meetings. After coding is complete, 1 analyst will examine codes using queries and matrices functions in Nvivo 10. The analyst and coding team will continue to meet regularly to discuss themes and data interpretation. Data interpretation will include situating findings within existing knowledge or theory related to risks for severe hypoglycemia. The involvement of multiple analysts and review of findings by a multidisciplinary team will be used as a check against interpretive bias [81,82,84,88,89]. A detailed audit trail will be maintained to record all decisions made throughout the analysis process.

We will obtain basic demographic characteristics and clinical information related to the patients’ diabetes history (including prior episodes of severe hypoglycemia) and health by retrospective review of the EHR.

### Sample size

We expect to enroll 75 participants per arm, for a total enrollment of 150 participants. Because no previous trials have specifically evaluated the effect of community paramedicine or diabetes self-management education interventions on diabetes self-management skills as measured by the DSMQ, the study sample size is determined on the basis of the mean DSMQ score difference observed between patients with good glycemic control (HbA_1c_ ≤ 7.5%) and those with poor or moderate glycemic control (HbA_1c_ > 7.5%) in earlier studies [75,76]. To evaluate the efficacy of D-REM, we seek to detect an increase of 1.0 point or greater in DSMQ score at 1 month in the intervention group, given an SD of 1.5. Given an effect size of 0.67 (Cohen d; mean [SD] difference in mean changes, 1.0 [1.5] at both time points), this will provide our study with 82% power at a significance level of.05, assuming a 20% dropout rate. Sample size was estimated via a mean change in repeated measures (pre-post) study design using Power Analysis and Sample Size (PASS 2019) software.

### Monitoring

No data and safety monitoring committee is planned for the study, which was determined to be minimal risk. The principal investigator and study coordinators will be responsible for monitoring study conduct and data quality, including accuracy of the data entered in REDCap. The IRB will review the trial protocol, consent documents, and other study-related materials annually per institutional policy. The study sponsor (NIDDK) receives annual progress reports but does not participate in audit procedures.

## Discussion

Community paramedicine interventions have emerged as a promising modality for enhancing diabetes care [59] given the complexity of self-management and the substantial economic impact of diabetes [83]. This study is important because it evaluates the efficacy of CP programs in improving diabetes care in patients who have experienced hypoglycemic episodes, a domain that is relatively understudied. A critical element of our study is its emphasis on understanding patient experiences, attrition rates, and barriers to participation. Insights gleaned from this study will inform enhancements in CP intervention strategies, making them more effective and acceptable. Study results will be disseminated in peer-reviewed publications and conference presentations, shared with clinicians and patients within our health system using available communication channels. The findings will have the potential to influence national diabetes care standards, advocate for the expansion of CP programs, and enhance the delivery of health care resources directly to patients’ homes, thereby complementing care delivered in traditional health care settings.

## Supporting information

S1 Appendix Appendix. (PDF)

S2 File Study Protocol. (PDF)

S1 Checklist MCHS2024–019 SPIRIT_Fillable-checklist. (DOC)

## Abbreviations

ANCOVA: analysis of covariance
ANOVA: analysis of variance
CP: community paramedic
DDS: Diabetes Distress Scale
D-REM: Diabetes Rescue, Engagement, and Management
DSMES: diabetes self-management education and support
DSMQ: Diabetes Self-Management Questionnaire
ED: emergency department
EHR: electronic health record
EMS: emergency medical services
EQ-5D-5L: 5-level EQ-5D version
HbA_1c_: hemoglobin A_1c_
IRB: institutional review board
MCHS: Mayo Clinic Health System
RCT: randomized clinical trial
