# Correction: Diabetes rescue, engagement, and management (D-REM) for hypoglycemia: Clinical trial protocol of a community paramedic program to improve diabetes management among adults with severe hypoglycemia

**DOI:** 10.1371/journal.pone.0336047

**Published:** 2025-11-03

**Authors:** Sumit Bhagra, Allison L. Ducharme-Smith, Michael B. Juntunen, Chad P. Liedl, Elizabeth H. Golembiewski, Wendy J. Sundt, Tami S. Krpata, Michelle A. Lampman, Anna L. Espinoza, Rozalina R. McCoy

The tenth author’s name is spelled incorrectly. The correct name is: Rozalina G. McCoy.
