# Supplementary material for: Diabetes rescue, engagement, and management (D-REM) for hypoglycemia: Clinical trial protocol of a community paramedic program to improve diabetes management among adults with severe hypoglycemia
Source: PLoS One. 2025 Jun 9;20(6):e0322177. doi: 10.1371/journal.pone.0322177 (PMC12148191; doi:10.1371/journal.pone.0322177)
Supplement: S1 Appendix — (PDF) [file pone.0322177.s001.pdf]

Mayo Clinic: Office for Human Research Protection

Oral Consent Script – Patient Participants

Protocol Title: Community Paramedicine Program to Improve Diabetes Management Among Adults Experiencing Severe Hypoglycemia
IRB #: 20-006799
Principal Investigator: Rozalina McCoy, MD MS

You are being asked to participate in a research study about patient experiences with and perceptions about very low blood sugar levels in diabetes, also called hypoglycemia. You are being invited to participate because you may have experienced an episode of hypoglycemia in the past.

If you agree to participate you will be asked to participate in an interview, which we expect to last approximately an hour or less. The interview will take place at a time that is convenient for you with a Mayo Clinic researcher over Zoom or Skype or telephone, depending on your preference. You will receive $25 as payment for your participation.

The interview will be audio-recorded and transcribed so that our study team can review an accurate record of your responses. Names will be removed from the transcripts, and your identity will not be revealed in the study results. Your medical record may also be accessed to collect information regarding your health care history and basic demographic information (age, sex, etc.) and information about your diabetes (diabetes type, medications you take, whether you had more than one episode of hyperglycemia or hypoglycemia that had to be treated in the emergency department or hospital, etc.).

If you decide to participate, you will need to read and sign the Authorization to Use and Disclose Protected Health Information (HIPAA) form. We are not allowed to use your protected health information without your signature on the HIPAA form. An extra copy is included for your records.

The risks associated with this research study are minimal, which means that we do not believe that they will be any different than what you would experience at a routine clinical visit or during your daily life. You may choose not to answer any questions that make you feel uncomfortable.

Although there is no direct benefit to you, there is a potential benefit to people in the future as a result of what we learn in this research study.

Your information or biospecimens collected as a part of this research will not be used or distributed for future research, even if identifiers are removed.

Please understand your participation is voluntary and you have the right to withdraw your consent or discontinue participation at any time without penalty. Specifically, your current or future medical care at the Mayo Clinic will not be jeopardized if you choose not to participate.

If you have any questions about this research study you can contact me [name of person doing the consent] at [phone number]. If you have any concerns, complaints, or general questions about

Master copies are retained online. Printed copies are considered current only on the date printed, unless stamped ORIGINAL or COPY in red ink.
© 2025 Mayo Foundation for Medical Education and Research

research or your rights as a participant, please contact the Mayo Institutional Review Board (IRB) to speak to someone independent of the research team at 507-266-4000 or toll free at 866-273-4681.

Master copies are retained online. Printed copies are considered current only on the date printed, unless stamped ORIGINAL or COPY in red ink.
© 2025 Mayo Foundation for Medical Education and Research
