# Supplementary material for: Diabetes rescue, engagement, and management (D-REM) for hypoglycemia: Clinical trial protocol of a community paramedic program to improve diabetes management among adults with severe hypoglycemia
Source: PLoS One. 2025 Jun 9;20(6):e0322177. doi: 10.1371/journal.pone.0322177 (PMC12148191; doi:10.1371/journal.pone.0322177)
Supplement: S2 File — (PDF) [file pone.0322177.s002.pdf]

IRB Minimal Risk Protocol Template

Note: If this study establishes a human specimen repository (biobank) for research purposes, do not use this template. Use the Mayo Clinic Human Specimen Repository Protocol Template found on the IRB home page under Forms and Procedures at http://intranet.mayo.edu/charlie/irb/

First-time Use: Use this template to describe your study for a new IRB submission.
1. Complete the questions that apply to your study.
2. Save an electronic copy of this protocol for future revisions.
3. When completing your IRBe application, you will be asked to upload this document to the protocol section.

Modification: To modify this document after your study has been approved:
1. Open your study in IRBe. Click on the study 'Documents' tab and select the most recent version of the protocol. Save it to your files.
2. Open the saved document and activate "Track Changes".
3. Revise the protocol template to reflect the modification points, save the template to your files
4. Create an IRBe Modification for the study and upload the revised protocol template.

General Study Information

Principal Investigator: Rozalina McCoy, MD, MS

Study Title: Community Paramedicine Program to Improve Diabetes Management Among Adults Experiencing Severe Hypoglycemia

Protocol version number and date: Version 3; September 19, 2022**Research Question and Aims**

HYPOTHESIS: Patients with diabetes who are enrolled in Diabetes-REM (Rescue, Engagement, and Management), a comprehensive community paramedicine management program, after an episode of severe hypoglycemia will have improved diabetes self-management (primary outcome), fewer episodes of levels 2 and 3 hypoglycemia and severe hyperglycemia, lower diabetes distress, and better quality of life.

AIMS, PURPOSE, OR OBJECTIVES:
The overall objective of this study is to assess the feasibility, efficacy, and acceptability of **Diabetes-REM** (Rescue, Engagement, and Management) to improve diabetes self-management among adults in Southeast Minnesota (SEMN) who have experienced severe hypoglycemia.

AIM 1: Evaluate the feasibility and efficacy of Diabetes-REM to improve diabetes self-management among adults with diabetes (type 1 and type 2) who experienced severe hypoglycemia.

Hypothesis 1: Diabetes-REM participants will have improved diabetes self-management (measured by the Diabetes Self-Management Questionnaire), as compared to controls. We will conduct a two-arm randomized controlled trial among 150 adults with diabetes in SEMN who have experienced severe hypoglycemia (level 3 hypoglycemia). Level 3 hypoglycemia is defined as any episode of hypoglycemia, documented or self-reported, that required third-party assistance to treat or terminate. This includes patients who were treated for

Published under a CC BY license, with permission from Mayo Foundation for Medical Education and Research, original copyright 2025.

hypoglycemia by caregivers, emergency medical services (EMS), or other healthcare providers (in the office, emergency department, hospital). The intervention group will be enrolled in Diabetes-REM for approximately 1 month, focused on identifying and addressing hypoglycemia risk factors and barriers to optimal diabetes care. Both groups will receive written information about diabetes, hypoglycemia, and clinic/community resources. Secondary outcomes will be self-reported glucose <54 mg/dL or hypoglycemia requiring 3rd party assistance, glucose ≥250 mg/dL or ketoacidosis, diabetes distress (Diabetes Distress Survey), and quality of life (EQ-5D). Outcomes will be assessed using electronic health records and surveys at baseline (prior to starting D-REM), month 1 (right after completing D-REM), and month 4 (3 months after completing D-REM). Feasibility metrics will focus on rates of screening, recruitment, randomization, retention, adherence, fidelity, and assessments.

AIM 2: Identify features of Diabetes-REM that were most meaningful to enrolled participants.
Hypothesis 2: Participants' perceptions of Diabetes-REM, gathered through semi-structured interviews of approximately 16 participants, will reveal whether/how specific program components served to meet their needs. This knowledge will facilitate iterative program improvement in preparation for the R01-funded efficacy/implementation trial.

AIM 3: If an eligible patient is not interested in participating in the Diabetes-REM intervention we would like to understand the reasons for why, as well as better understand patient experience with and perceptions of hypoglycemia.
Hypothesis 3: Understanding the reasons for why patients are not interested in participating in the Diabetes-REM intervention will help inform how this program should be improved before it is implemented in the practice. Patient's experience with hypoglycemia can also inform program components and implementation.

BACKGROUND (*Include relevant experience, gaps in current knowledge, preliminary data, etc.*):

Diabetes is one of the most common and costly chronic diseases in the U.S. affecting more than 30 million people, or 9.3% of the U.S. population.[1] It negatively impacts patient health and quality of life (QoL), and puts financial and logistical strain on the U.S. health care system.[2-4] The goal of glucose-lowering therapy is to prevent acute and chronic diabetes complications by controlling hyperglycemia while avoiding hypoglycemia.[5-10] Preventing hypoglycemia, particularly severe hypoglycemia, is a key component of diabetes management. Prior studies, including our research,[11,12] have linked severe hypoglycemia to cardiovascular events,[13-17] mortality,[11,13,15-22] decreased QoL,[12] disability,[23] dementia,[24-26] and increased healthcare costs.[23] Clinical guidelines explicitly recommend screening at-risk patients for severe hypoglycemia, eliciting hypoglycemia risk factors, and taking steps to address these risk factors in order to prevent hypoglycemia recurrence.[5]

However, our research revealed gaps in identifying patients who experience hypoglycemia and preventing its recurrence. Retrospective analysis of diabetes-focused visits in primary care and endocrinology practices revealed that at-risk adults (i.e. those treated with insulin or sulfonylurea) were screened for hypoglycemia in 72% of endocrinology and 47% of primary care visits.[27] After patients reported hypoglycemia, treatment was de-intensified in just 46% of endocrinology and 30% of primary care visits; education about hypoglycemia was provided in 42% of endocrinology and 35% of primary care visits; and glucagon was prescribed in 4% of endocrinology and 9% of primary care visits. We found similar low rates of treatment modification among 5,721 adults with diabetes hospitalized for hypoglycemia using OptumLabs Data Warehouse (OLDW), a de-identified administrative claims database of commercially-insured and Medicare Advantage beneficiaries across the U.S.[28,29] There was no increase in glucose monitoring, glucagon fills, or hemoglobin A_{1c} (HbA_{1c}) levels in the 6-months after compared to 6-months before their hospitalization (under peer-review). These data reinforce

Published under a CC BY license, with permission from Mayo Foundation for Medical Education and Research, original copyright 2025.

the many missed opportunities to identify at-risk patients, elucidate hypoglycemia risk factors, and intervene on these risk factors to prevent recurrent hypoglycemic events.

As a result, severe hypoglycemia continues to cause major harm to patient health and rates of severe hypoglycemia remain unacceptably high.[30-32] We recently estimated that at least 9,500 emergency department (ED) visits and hospitalizations for hypoglycemia may have been avoided across the U.S. over a 2-year period if patients were treated less intensively.[33] The CDC estimates that in 2015 (last year with available data) there were 13.8 ED visits and 2.6 hospitalizations for severe hypoglycemia, respectively, per 1,000 diabetic adults in the U.S.[34] In our analyses using OLDW, the overall rate of ED/hospital utilization for hypoglycemia was 9.1 per 1,000 persons/year, with higher rates among patients with type 1 diabetes (32.1 vs. 8.2 per 1000 persons/year with type 2 diabetes), lower annual household income (12.3 vs. 4.5 per 1,000 persons/year with <$40,000 vs. ≥$100,000), and multiple comorbidities (57.4 vs. 3.5 per 1000 persons/year in the presence of ≥8 vs. ≤1 comorbidities).[35] These findings demonstrate that preventing hypoglycemia requires a comprehensive approach, which considers the totality of the patient's clinical and social needs in addition to those posed by diabetes. Such comprehensive management is not part of current care delivery models.

The vast majority of severe hypoglycemic events are treated by caregivers at home, with some culminating in an emergency medical services (EMS) call. Approximately half of hypoglycemic events managed by EMS are treated on scene without transport to the ED or hospital.[36-38] We previously examined EMS calls for hypoglycemia in Olmsted County, MN between 2003-2009 (1,473 calls by 914 individuals),[39-42] and found that 60% were transported to the ED while half were hospitalized.[42] Hypoglycemic events among non-transported patients are likely to be missed by the healthcare team and not intervened upon, particularly as patients do not routinely notify clinicians about hypoglycemic events[43,44] and clinicians do not screen at-risk patients for hypoglycemia.[27] Prior hypoglycemia is one of the strongest risk factors for future hypoglycemia.[35,45-50] Identifying and engaging patients treated for hypoglycemia by caregivers and by EMS is a logical next step toward a comprehensive hypoglycemia screening and management strategy for highest risk individuals.[51]

Thus, there is great need for comprehensive, accessible, and sustainable interventions to improve diabetes self-management and prevent hypoglycemia among patients with diabetes. In this application, we will examine the feasibility, efficacy, and acceptability of Diabetes-REM to improve diabetes self-management among people with diabetes who experienced severe (level 3) hypoglycemia. This work will provide the preliminary data needed for a multi-center, multi-region R01-funded trial of Diabetes-REM to improve diabetes management, prevent severe hypoglycemia, and improve health outcomes among people with diabetes with, or at increased risk for, severe hypoglycemia.

Community Paramedicine (CP) has emerged across the U.S. as a scalable, effective, and efficient care delivery model to improve health care access for underserved communities and populations[52-59]. In contrast to traditional emergency response services, CPs focus on preventive care with emphasis on primary care delivery, education, prevention, and wellness[57-63]. There are two core attributes of community paramedicine that make it uniquely suited to meet the multi-faceted needs of rural and underserved communities[64-66]. The primary healthcare model focuses on preventing hospital or emergency department (ED) admissions/readmissions and monitoring chronic illness[54,59,67-69]. The community coordination model seeks to connect patients to appropriate social, community, and medical services[58,59]. Thus far, most CP programs have focused on specific high risk patient populations, most often those with history of frequent hospital, ED, and/or emergency medical services (EMS) utilization, multi-morbidity, and frailty[54,59,67,69-73]. We will build on this work to specifically target high-risk adults with

Published under a CC BY license, with permission from Mayo Foundation for Medical Education and Research, original copyright 2025.

diabetes, seeking not only to reduce acute care utilization but to also improve self-management, glycemic control, and quality of life.

Mayo Clinic Ambulance launched its CP program in 2015. In a pilot study carried out in Barron County (rural Northwest Wisconsin) in 2016, 2 CPs took care of 42 patients referred due to high rates of healthcare utilization. CPs saw patients for 1-hr home visits (1-2 times per week), during which they performed health assessment, medication review, physical examination, environment assessment, and carried additional orders if requested by the patient's primary care physician. Primary care, ED, and hospital utilization decreased by 53%, 59%, and 60%, respectively, in the 6 months after compared to 6 months before the intervention. Our proposed work will build on this effort to engage patients with uncontrolled diabetes and improve diabetes-specific and overall health outcomes.

Study Design and Methods

METHODS

Aim 1: This pilot study will evaluate Diabetes-REM through a two-group, parallel arm randomized controlled trial (RCT) carried out in the community setting across 5 counties in SEMN (Freeborn, Mower, Olmsted, Steele, Wabasha). The intervention group will receive CP home visits and telephone calls for approximately 1 month (tailored to the patient's clinical situation and need). Both groups will receive printed diabetes education materials and a resource guide for contacting their diabetes care team in addition to usual care. Aim 1 will assess program feasibility and efficacy, with the primary outcome of diabetes-self management, measured by the Diabetes Self-Management Questionnaire (DSMQ).[74] DSMQ was chosen because its 5 subscales capture the wide range of factors that impede optimal diabetes care and may contribute to hypoglycemia risk (i.e. dietary control, medication adherence, blood glucose monitoring, physical activity, and physician contact). Secondary outcomes will include recurrent hypoglycemia, severe hyperglycemia, diabetes distress, and health-related QoL.

Study population: We will identify, consent, and enroll adults with diabetes who experienced level 3 hypoglycemia and live in Freeborn, Mower, Olmsted, Steele, or Wabasha counties. These areas have high prevalence of diabetes, few diabetes providers, and an established MCAS infrastructure.

Eligible participants will be identified using the following complementary methods:
1. Patients who are treated for hypoglycemia by MCAS. The MCAS electronic health record system, Tablet (Zoll product) will be used to generate a report of events for hypoglycemia, identified using the discrete fields of glucose value on the scene (set to <54 mg/dL to identify level 2 hypoglycemia) and medication administration (set to glucose, D10W, D25W, D50W, or glucagon). A real-time report will be generated by FirstWatch based on these criteria and emailed to the study team. The report contains information about the ambulance encounter, including patient name, date of birth, and address. This information will be used to identify the individual's MRN and screen them for eligibility for the study.

2. Patients who were treated in Mayo Clinic ED or hospital for hypoglycemia. We will use the electronic health record (EHR) to identify patients with a ICD10 code for hypoglycemia (using a validated code set used extensively in our research) present on an ED claim or on a hospital claim on the day of either hospital admission or discharge. These charts will be reviewed weekly to identify patients meeting eligibility criteria.

3. Patients who have a diagnosis of hypoglycemia on their electronic health record problem list.

Published under a CC BY license, with permission from Mayo Foundation for Medical Education and Research, original copyright 2025.

4. Patients who experienced level 3 hypoglycemia and are referred to the community paramedic service by their healthcare provider.

Enrollment & consent: A member of the study team will verify inclusion/exclusion criteria using the electronic health record (EHR) and contact eligible individuals to describe the study and offer enrollment either by mail or electronically.

If the patient is here at Mayo Clinic they may be approached for study participation in-person.

Up to five contact attempts will be made to contact the patient. If there is no response to the first phone call attempt, to maximize the likelihood of patient contact, potential participants will simultaneously be contacted by online portal (if they have an active portal account) and by mail, with the recruitment letter briefly describing the study and asking potential participants to call the study coordinator back if interested.

During the initial phone call, the team member will explain the study and intervention to the participant and confirm eligibility criteria per pre-defined inclusion/exclusion criteria. If the individual is interested in enrolling in the study, a consent form will be offered to the patient by mail or electronically e-mailed, along with the initial (baseline) questionnaire which will also be offered by mail or electronically e-mailed. If a subject is able to electronically sign the consent form, the initial baseline questionnaire can be done with the study coordinator immediately over the phone. Subsequent to receipt of the signed consent form and survey, participants will be randomized to intervention vs. control arms. Randomization will be blocked by diabetes type (type 1 vs. type 2) and how the hypoglycemic event was managed (caregivers or bystander without calling for medical attention vs. EMS only vs. ED/hospital). Participants will be sequentially recruited until the target accrual of 150 participants (75 per arm) is reached.

Once the consent form is returned, patient contact information will be communicated to the CP team, who will arrange further contact as part of the intervention.

The two study arms will be:
1. Intervention: CP program + education materials
2. Control: usual care + education materials

*Intervention (**Figure 1**)*: CP will meet consented patients at their home or alternate mutually-agreeable patient-requested location within 5 business days. Masking during in-home patient visits will follow contemporaneous Mayo Clinic policies. Patients will be seen for approximately 1-hr for in-person evaluation and follow-up will be determined on an individual basis based on patient need. We estimate an average of two 1-hour in-person visits and two 30-min phone visits per patient, but study CPs will be able to accommodate more frequent visits if required per patient's clinical need.

During each visit, CPs will perform health status and social determinants of health assessments, physical exam, medication review/counseling, and goal setting/review. Guided by participant's needs, CPs can engage clinical resources (e.g. primary care, pharmacy, social services), activate community resources (e.g. meal delivery, food bank, social support), deliver hands-on education (e.g. take participant grocery shopping to teach about healthy diet), or carry out clinician orders if part of usual care (e.g. vital sign monitoring, obtain urine/blood samples). Bidirectional communication with the primary care team will occur via Epic. CPs will be supported by a clinical

Published under a CC BY license, with permission from Mayo Foundation for Medical Education and Research, original copyright 2025.

resource team (Drs. McCoy, Bhagra & Ms. Kasper), who will be available for ongoing questions. Dr. McCoy (PI) will also communicate with the CPs regularly to help address any questions or concerns that participants might have.

Participants will be enrolled for approximately one month. Upon completion of the pilot program, all participants will be transitioned to their primary care team. The study team will communicate directly with the primary care team to ensure continuity of care.

Education materials: Upon enrollment, study staff will provide each participant with a diabetes resource card, which will provide a list of clinic and community-based diabetes resources. Clinic resources include information about different care team members (e.g. primary care provider and nurse, pharmacist, social worker, diabetes nurse, endocrinologist, dietitian) and how to make an appointment to see them. Community resources include community health workers, dieticians (available in local grocery stores), family services, food banks, and regional Living Well with Chronic Conditions programing.[75] Participants will also be provided with a diabetes education packet, which will include information on diabetes self-management, healthy diet, physical activity, glucose self-monitoring, and hypoglycemia. Both materials will be developed by the study team, led by Ms. Johnson (education specialist) and Ms. Kasper, RN CDE. These materials will ensure that all participants receive the current standard of care after experiencing severe hypoglycemia.

Figure 1. Schematic of the Diabetes-REM intervention. SDOH, social determinants of health. PCP, primary care provider.

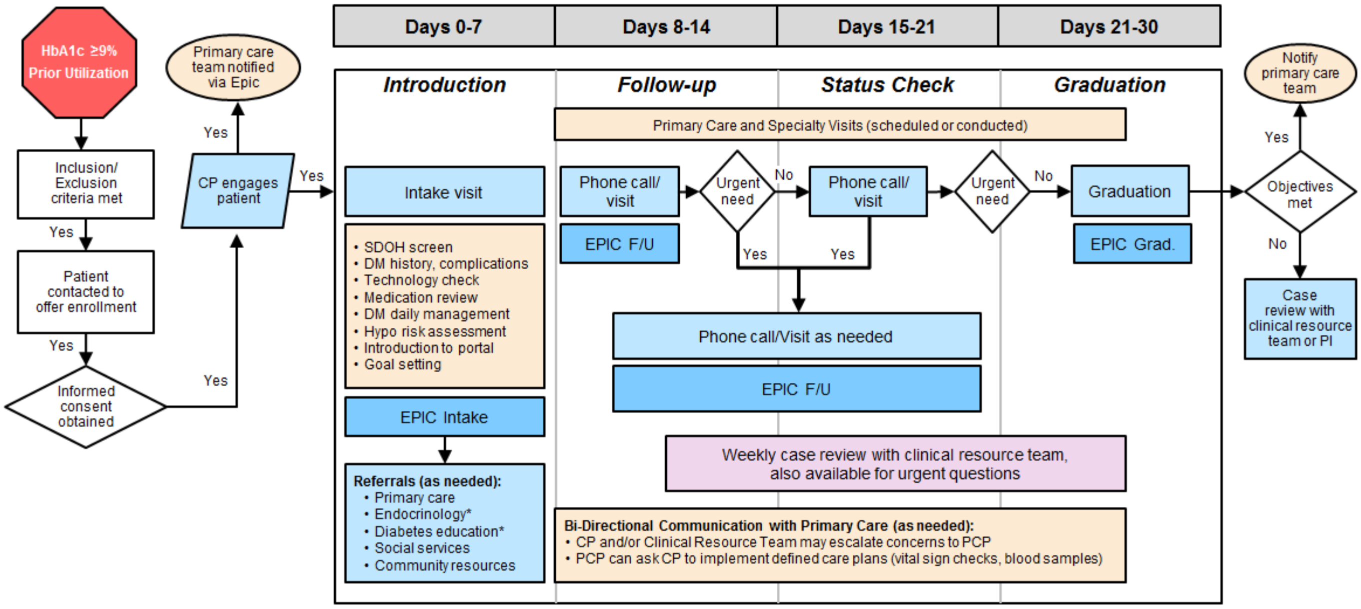

Program evaluation: Participant data will be collected at baseline (prior to the first CP visit), 1 month or right after completing the CP intervention (to assess immediate program effect), and 4 months or 3 months after completing the CP intervention (to assess short-term durability of program effect).

Published under a CC BY license, with permission from Mayo Foundation for Medical Education and Research, original copyright 2025.

The electronic health record (EHR) will be used to ascertain patient age, gender, race/ethnicity, rurality, medications, comorbidities, and prior EMS/ ED/ hospital utilization for hypo- and hyperglycemia. Diabetes type and duration will be ascertained using baseline survey. Primary and secondary endpoints will be measured via surveys conducted at baseline, 1 month, and 4 months. All surveys will either be mailed via US Postal Service once by the Mayo Clinic Survey Research Center or done in real time, over the phone, with the study coordinator. If a subject is not reached after two call attempts, for each survey time point, second mailings will go out to non-responders after 3 weeks. If participants do not respond to the 2nd mailing, they will be contacted by a study team member by phone, one last time, to remind them to complete the surveys. Additional copies will be mailed if necessary.

Survey will assess the primary outcome of diabetes-self management (DSMQ[74]), which has been associated with glycemic control[76] and microvascular complications.[77] Secondary outcomes, also ascertained via survey, include self-reported hypoglycemia (glucose <54 mg/dL or need for 3rd party assistance), hyperglycemia (glucose ≥250 mg/dL), diabetes distress (Diabetes Distress Scale[78,79]), and QoL (EQ-5D[80,81]). EHR will be used to identify EMS/ ED/ hospital utilization for hypo-and hyperglycemia during the study period.

Aim 2: Identify features of Diabetes-REM that were most meaningful to enrolled participants through participant interviews. This will allow us to better understand whether/how specific program components (e.g. home visit, environmental/ behavioral assessment, active learning) addressed participant's needs related to managing their diabetes.

We will use *stratified purposeful sampling* to intentionally select approximately 16 participants to represent differing demographic, clinical, and geographic subgroups.[82,83] We will contact participants who completed Diabetes-REM by phone and continue enrollment until thematic saturation in their interview responses is reached.[84-86]

All information connecting study ID numbers to participant names will be securely kept and destroyed after completion of the project. Five years after publication of the results (per standard journal guidelines), all paper copies of the forms, as well as the database used for analysis, will be destroyed.

Aim 3: Participants eligible for Aim 1 who decline participation in the Diabetes-REM study will be invited to participate in a one-time semi-structured qualitative interview conducted by telephone or videoconference technology, depending on participant preference. We will recruit participants until we reach thematic saturation. We are anticipating interviewing a minimum of 16 patients up to 25 patients. Participants will be recruited during the initial contact if they decline participation in the trial (Aim 1). Interviews will be conducted by telephone or videoconferencing technology, recorded, and professionally transcribed.

The purpose of this qualitative aim is to explore and understand patient perceptions of severe hypoglycemia risk factors, experiences, and precipitating factors; understand what services or resources can help prevent hypoglycemia; and understand why they were not interested in the Diabetes-REM intervention. Semi-structured interview guides will include open-ended questions in the following domains: 1) participants' perception of the patient's severe hypoglycemia risk; 2) what they believe caused their prior hypoglycemic events or predisposed them to experiencing hypoglycemia; 3) how serious they believe these events to be; 4) what interventions, treatment regimen changes, and/or support systems could have prevented their earlier events and may help prevent future events; 5) what were the reasons for them declining participation in Diabetes-REM; and 6) what could be improved or changed about the Diabetes-REM program if it were to be incorporated into clinical practice. The purpose of the patient questions is to understand whether patients view themselves as at risk, what factors inform their assessments of risk, and what can be done to lower their risk. These perspectives will help

Published under a CC BY license, with permission from Mayo Foundation for Medical Education and Research, original copyright 2025.

us determine what patient-reported factors need to be considered in designing interventions to reduce the risks of hypoglycemia for patients with diabetes.

Recruitment: Participants will be identified as part of Aim 1 and introduced to this interview study if they decline participation in Diabetes-REM. Oral consent will be obtained for participation in the study. A consent form will be offered to the patient by mail or electronically e-mailed. Participants will receive $25 in remuneration for taking part in AIM 3 of the study.

Interviews will follow a semi-structured interview guide. Interviews will last 45-60 minutes and be conducted by a member of the study team with experience in qualitative interviews. Interviews will occur by telephone or videoconference technology, depending on participant preference. Interviews will be scheduled for a time convenient for the participant.

Participant demographic information will be collected through the interviews and by electronic health record review (patient age, sex, ethnicity/race; patient's diabetes type and duration, HbA_{1c}, diabetes medications, history of severe hypoglycemia). All interviews will be audio recorded and transcribed verbatim by a trained transcriptionist.

Data analysis: Qualitative assessment of individual interviews will use a conventional or inductive content analysis approach,[87] which is appropriate when the existing literature is limited. Data analysis will be concurrent with data collection. Qualitative analysis will begin with two study team members reviewing each transcript several times and making notes of their initial impressions and descriptions of the data (analytic memos). Transcripts will be imported into NVivo 10 software. Two coders will begin coding data line-by-line to generate a codebook. Coders will meet weekly to discuss emerging codes. The codebook will be refined during meetings. Once coding is complete, one analyst will examine codes using queries and matrices functions in Nvivo 10. The analyst and coding team will continue to meet regularly to discuss themes and data interpretation. Data interpretation will include situating findings within existing knowledge or theory related to risks for severe hypoglycemia. The use of multiple analysts and review of findings by a multi-disciplinary team will be used as a check against interpretive bias.[88-92] An analysis audit trail will document decisions made during the analyses.

We will obtain basic demographic and clinical information related to the patients' diabetes history (including prior episodes of severe hypoglycemia) and health by retrospective review of the electronic medical records.

Subject Information

Target accrual is the proposed total number of subjects to be included in this study at Mayo Clinic. A "Subject" may include medical records, images, or specimens generated at Mayo Clinic and/or received from external sources.

Target accrual:

1. Aim 1: 150 participants (75 per study arm)
2. Aim 2: 16 participants
3. Aim 3: 16 - 25 participants

Subject population:

Published under a CC BY license, with permission from Mayo Foundation for Medical Education and Research, original copyright 2025.

AIM 1:
Inclusion criteria will be:
1. Experience of level 3 hypoglycemia (see criteria below).
2. Age ≥18 years
3. Type 1 or type 2 diabetes
4. Paneled to a Mayo Clinic or Mayo Clinic Health System practice
5. Able to provide informed consent
6. Community-dwelling in either Freeborn, Mower, Olmsted, Steele, or Wabasha County.

Exclusion criteria will be:
1. Cognitive impairment precluding informed consent
2. Lack of conversational English skills
3. Residency in a long-term care facility
4. Enrolled in hospice
5. Currently enrolled in a care coordination or active disease management program. Patients who were enrolled in those programs can be enrolled in the trial 30 days after their last care coordination encounter.
6. Advanced or terminal illness

Identifying patients who experienced hypoglycemia:
1. Treated by MCAS for hypoglycemia: Eligible participants will be identified using Mayo Clinic Ambulance call logs to identify encounters with glucose <54 mg/dL and/or treatment with glucose, dextrose (D10W, D25W, D50W), or glucagon in the prior week. A real-time report will be generated by FirstWatch based on these criteria and emailed to the study team. The report contains information about the ambulance encounter, including patient name, date of birth, and address. This information will be used to identify the individual's MRN and screen them for eligibility for the study.

2. Patients who were treated in Mayo Clinic ED or hospital for hypoglycemia. We will use the electronic health record (EHR) to identify patients with a ICD10 code for hypoglycemia (using a validated code set used extensively in our research[35]) present on an ED claim or on a hospital claim on the day of either hospital admission or discharge. These charts will be reviewed weekly to identify patients meeting eligibility criteria.

3. Patients who have a diagnosis of hypoglycemia on their electronic health record problem list.

4. Patients who experienced level 3 hypoglycemia and are referred to the community paramedic service by their healthcare provider.

During each data pull, we will cross-reference prior eligible patient lists to make sure that patients who previously refused participation in the trial will not be contacted for a second time. The only exception to this will be patients who experienced a new qualifying episode of hypoglycemia treated by EMS, ED, or hospital as the severity of these events may prompt them to reconsider their interest in participating in the study.

Published under a CC BY license, with permission from Mayo Foundation for Medical Education and Research, original copyright 2025.

Individuals meeting inclusion/exclusion criteria will be contacted by the study coordinator to describe the study and offer enrollment. An oral consent script and HIPAA authorization form will then be mailed to eligible participants who expressed interest in enrollment, along with the initial (baseline) questionnaire. Subsequent to receipt of the signed HIPAA authorization form and survey, participants will be randomized to intervention vs. control arms, blocked by diabetes type (type 1 vs. type 2) and how the hypoglycemic event was managed (by caregiver/bystander vs. EMS vs. ED/hospital). Participants will be sequentially recruited until the target accrual of 150 participants (75 per arm) is reached.

AIM 2:
We will reach out to participants who had been randomized to, and completed, the D-REM intervention. Participants will be purposefully sampled from among all participants who had been randomized to the Diabetes-REM intervention to represent a priori defined differing demographic, clinical, and geographic subgroups. We anticipate approximately 16 participants.

AIM 3:
Eligible participants who declined participation in Aim 1 will then be offered participation in the Aim 3 interview. Participants will be offered participation sequentially, until target accrual is reached. We anticipate approximately 16 participants but will recruit more if needed to reach thematic saturation.

Biospecimens

Not applicable

Review of medical records, images, specimens

Check all that apply (data includes medical records, images, specimens).

☐ Only data that exists before the IRB submission date will be collected.

Date Range for Specimens and/or Review of Medical Records:
Examples: *01/01/1999 through 12/31/2015*, or all records through *mm/dd/yyyy*.

Note: The Date Range must include the period for collection of baseline data, as well as follow-up data, if applicable.

☒ The study involves data that exist at the time of IRB submission **and** data that will be generated after IRB submission. Include this activity in the Methods section.
Examples
- The study plans to conduct a retrospective chart review and ask subjects to complete a questionnaire.
- The study plans to include subjects previously diagnosed with a specific disease and add newly diagnosed subjects in the future.

☐ The study will use data that have been collected under another IRB protocol. Include in the Methods section and enter the IRB number from which the research material will be obtained. *When appropriate, note when subjects have provided consent for future use of their data and/or specimens as described in this protocol.*

Published under a CC BY license, with permission from Mayo Foundation for Medical Education and Research, original copyright 2025.

Enter one IRB number per line, add more lines as needed

☐ Data ☐ Specimens ☐ Data & Specimens ______________________________________

☐ Data ☐ Specimens ☐ Data & Specimens ______________________________________

☐ Data ☐ Specimens ☐ Data & Specimens ______________________________________

Data Analysis

POWER STATEMENT:

This trial will randomize individuals to receive the Diabetes-REM (Rescue, Engagement, and Management) intervention or to continue with usual care. Both groups will receive written educational materials on diabetes and hypoglycemia, and a resource card about clinical and community resources available in their area.

Sample size: We expect to enroll 75 subjects per arm, for a total enrollment of 150 subjects. Because there have been no prior trials directly examining the impact of community paramedicine or diabetes self-management education interventions on diabetes self-management skills as measured by the Diabetes Self-Management Questionnaire (DSMQ), study sample size is calculated based on mean DSMQ score difference between patients with good glycemic control (hemoglobin A_{1c} (HbA_{1c}) ≤7.5%) compared to those with poor or average glycemic control (HbA_{1c} >7.5%) as found in previous studies.[93,94] To evaluate the efficacy of Diabetes-REM, we seek to detect an increase of 1.0 point or greater in DSMQ score at 1-month in the intervention group, given a standard deviation of 1.5. Given an effect size of 0.67 (Cohen's d; 1.0 difference in mean changes, SD=1.5 at both time points) this will provide our study with 82% power at a significance level of 0.05, assuming a 20% dropout rate. Sample size was estimated via a mean change in repeated measures (pre-post) study design using Power Analysis and Sample Size (PASS 2019) software.

Primary outcome: Analysis of covariance will be used to analyze the mean difference in score between arms at 1-month, adjusting for baseline scores. To assess changes from baseline at 1-month and 4-months we will utilize repeated measures ANOVA via a modified mixed model. This method produces the least biased results when conducting an intention-to-treat analysis and allows for missing data, unlike a standard ANCOVA. We will compare baseline demographics between arms using t-tests or the Kruskal-Wallis H test for continuous variables and chi-square tests or Fisher's exact test for categorical variables; characteristics imbalanced between arms at a $p<.05$ level will be included as covariates in the final model.

Secondary outcomes: Binary outcomes (self-report of hypoglycemia and hyperglycemia) will be analyzed using logistic regression to generate odds ratio with 95% confidence intervals for comparison between arms. Normally distributed continuous outcomes (Diabetes Distress Scale (DSS), EuroQol-5d (EQ-5D) will be examined for differences at the 1-month mark using paired t-tests. As we anticipate some sum and subscale scores to be non-parametric, those differences in scale scores at 1-month between arms will be analyzed using analysis of covariance, adjusting for baseline scores. As with our primary outcome, overall change from

Published under a CC BY license, with permission from Mayo Foundation for Medical Education and Research, original copyright 2025.

baseline at 1-month and 4-months will be explored using repeated measures ANOVA via a modified mixed model. Models will be adjusted for baseline characteristics as outlined above.

Aim 2 of the study is a qualitative assessment of participants' perceptions of Diabetes-REM and its impact on their diabetes management. Our objective is to gather a rich description of experiences and insights related the Diabetes-REM intervention from study participants. As such, sampling is guided by the principle of *qualitative clarity* as an analog to the quantitative concept of statistical power.[86] We will use *stratified purposeful sampling* to intentionally select participants to represent predefined and explicit traits or conditions.[86,87] This will ensure that we get a rich description across patients of different demographics and clinical profiles. Our *a priori* objective is to capture the perspectives of participants with type 1 and type 2 diabetes, younger and older age, those referred from the outpatient setting, transported to the emergency department, and those treated by EMS without transport, and those living in a rural area and in a more urban setting. While we anticipate that approximately 16 individuals would allow us to sample a representative population, the ultimate sample size will depend on the number needed to inform the key elements of the phenomenon studied.[90] Thus, when no new concepts or themes emerge from the interviews, *data saturation* will be reached. To determine when data saturation occurs, analysis will occur concurrently and iteratively with data collection.[90] Participants will be recruited until information redundancy (thematic saturation) in their interview responses is reached.[88,89]

Aim 3 of the study is a qualitative assessment of participant's perceptions of hypoglycemia and services/programs to reduce hypoglycemia risk. We expect to reach thematic saturation with 16 participants. To determine when data saturation occurs, analysis will occur concurrently and iteratively with data collection.[90] Participants will be recruited until information redundancy (thematic saturation) in their interview responses is reached.[88,89]

DATA ANALYSIS PLAN:

Endpoints

Primary: diabetes-self management (measured by the validated Diabetes Self-Management Questionnaire [DSMQ][74])

Secondary: (1) diabetes distress, measured by the validated Diabetes Distress Scale (DSS);[78,79] (2) health-related quality of life (measured using the EQ-5D) [80,81]; (3) self-reported hypoglycemia (level 2 hypoglycemia defined as glucose <54 mg/dL or level 3 hypoglycemia defined by need for 3rd party assistance); (4) self-reported severe hyperglycemia (glucose ≥250 mg/dL); (5) EMS/ ED/ hospital utilization for hypoglycemia and hyperglycemia during the study period.

Statistical Analysis

AIM 1. Analysis for all participants will occur in the arm in which they were assigned, in accordance with intention-to-treat guidelines. Descriptive statistics will be summarized using mean and standard deviation for continuous variables and frequency and percentages for categorical variables. We will assess any potential imbalances in baseline characteristics between arms using t-tests or the Kruskal-Wallis H test for continuous variables and chi-square tests or Fisher's exact test for categorical variables. The primary outcome of this aim, change in DSMQ score from baseline to 1 month, will be analyzed by using analysis of covariance (ANCOVA), adjusting for baseline patient scores. Overall change in DSMQ from baseline to 4 months will be assessed using repeated measures analysis of variance (ANOVA) via a modified mixed model. Secondary outcomes will be

Published under a CC BY license, with permission from Mayo Foundation for Medical Education and Research, original copyright 2025.

analyzed for differences at 1 month using paired t-tests for normally distributed data, and ANCOVA for non-parametric data. For binary outcomes, we will perform logistic regression adjusting for baseline characteristics.

AIM 2. In this qualitative aim we will use in-depth interview method using semi-structured interview guides to explore and understand participant perceptions of Diabetes-REM and its impact on diabetes management. These perspectives will also help us identify areas for program improvement.

Interviews will last 45-60 minutes and be conducted by an experienced qualitative interviewer via telephone. All interviews will be audio recorded and transcribed verbatim by a trained transcriptionist.

Data analysis: Qualitative assessment of individual interviews will use a thematic approach.[93,94] Preliminary analysis of the data will be concurrent with data collection in order to determine when saturation has been reached.[84-86] Two study members will review each transcript several times and make notes of their initial impressions and descriptions of the data (analytic memos). Those notes will be used to generate labels or codes, which will be arranged into higher order categories to represent key themes related to the study aims. To facilitate data queries for analysis, a coding framework will be applied to transcripts by at least two trained coders who will discuss and come to agreement prior to entering data into NVivo software. Data interpretation will include situating findings within existing knowledge or theory related to diabetes self-management and risks of hypoglycemia. The use of multiple analysts and review of findings by a multi-disciplinary team will be used as a check against interpretive bias.[88-92] An analysis audit trail will document decisions made during the analyses.

AIM 3. In this qualitative aim we will use in-depth interview method using semi-structured interview guides to explore and understand participant perceptions of hypoglycemia and programs/services that may reduce risk of hypoglycemia. These perspectives will also help us identify areas for program improvement.

Interviews will last 45-60 minutes and be conducted by an experienced qualitative interviewer via telephone or videoconference. All interviews will be audio recorded and transcribed verbatim by a trained transcriptionist.

Data analysis: Qualitative assessment of individual interviews will use a conventional or inductive content analysis approach,[87] which is appropriate when the existing literature is limited. Data analysis will be concurrent with data collection. Qualitative analysis will begin with two study team members reviewing each transcript several times and making notes of their initial impressions and descriptions of the data (analytic memos). Transcripts will be imported into NVivo 10 software. Two coders will begin coding data line-by-line to generate a codebook. Coders will meet weekly to discuss emerging codes. The codebook will be refined during meetings. Once coding is complete, one analyst will examine codes using queries and matrices functions in Nvivo 10. The analyst and coding team will continue to meet regularly to discuss themes and data interpretation. Data interpretation will include situating findings within existing knowledge or theory related to risks for severe hypoglycemia. The use of multiple analysts and review of findings by a multi-disciplinary team will be used as a check against interpretive bias.[88-92] An analysis audit trail will document decisions made during the analyses.

We will obtain basic demographic and clinical information related to the patients' diabetes history (including prior episodes of severe hypoglycemia) and health by retrospective review of the electronic medical records.

REFERENCES

Published under a CC BY license, with permission from Mayo Foundation for Medical Education and Research, original copyright 2025.

1. *Centers for Disease Control and Prevention. National Diabetes Statistics Report, 2017*. 2017. https://www.cdc.gov/diabetes/data/statistics/statistics-report.html
2. Huang ES, Brown SE, Ewigman BG, Foley EC, Meltzer DO. Patient perceptions of quality of life with diabetes-related complications and treatments. Research Support, N.I.H., Extramural
Research Support, U.S. Gov't, P.H.S. *Diabetes Care*. Oct 2007;30(10):2478-83. doi:10.2337/dc07-0499
3. American Diabetes Association. Economic Costs of Diabetes in the U.S. in 2012. *Diabetes Care*. Mar 6 2013;36(4):1033-1046. doi:10.2337/dc12-2625
4. CDC. Centers for Disease Control and Prevention. National Diabetes Statistics Report: Estimates of Diabetes and Its Burden in the United States. National Center for Chronic Disease Prevention and Health Promotion, Division of Diabetes Translation. U.S. Department of Health and Human Services. Accessed May 23, 2016. www.cdc.gov/diabetes/pubs/statsreport14/national-diabetes-report-web.pdf
5. American Diabetes Association Standards of Medical Care in Diabetes—2020. Section 6. Glycemic Targets. *Diabetes Care*. 2020;43(Supplement 1):S66-S76. doi:10.2337/dc20-S006
6. NICE. National Institute for Health and Care Excellence Pathways: Managing Blood Glucose in Aults with Type 2 Diabetes. National Institute for Health and Care Excellence. April 23, 2019. Updated March 26, 2019. Accessed April 23, 2019. https://pathways.nice.org.uk/pathways/type-2-diabetes-in-adults
7. Garber AJ, Abrahamson MJ, Barzilay JI, et al. Consensus Statement By The American Association Of Clinical Endocrinologists And American College Of Endocrinology On The Comprehensive Type 2 Diabetes Management Algorithm – 2019 Executive Summary. *Endocrine Practice*. 2019;25(1):69-100. doi:10.4158/cs-2018-0535
8. Qaseem A, Wilt TJ, Kansagara D, et al. Hemoglobin A1c Targets for Glycemic Control With Pharmacologic Therapy for Nonpregnant Adults With Type 2 Diabetes Mellitus: A Guidance Statement Update From the American College of PhysiciansACP Guidance Statement on HbA1c Targets With Pharmacologic Therapy. *Annals of Internal Medicine*. 2018;168(8):569-576. doi:10.7326/m17-0939
9. *VA/DoD Clinical Practice Guideline for the Management of Type 2 Diabetes Mellitus in Primary Care*. Defense DoVAaDo; 2017. April 2017.
10. Davies MJ, D'Alessio DA, Fradkin J, et al. Management of Hyperglycemia in Type 2 Diabetes, 2018. A Consensus Report by the American Diabetes Association (ADA) and the European Association for the Study of Diabetes (EASD). *Diabetes Care*. 2018:dci180033. doi:10.2337/dci18-0033
11. McCoy RG, Shah ND, Van Houten HK, Wermers RA, Ziegenfuss JY, Smith SA. Increased mortality of patients with diabetes reporting severe hypoglycemia. Article. *Diabetes Care*. 2012;35(9):1897-1901. doi:10.2337/dc11-2054
12. McCoy RG, Van Houten HK, Ziegenfuss JY, Shah ND, Wermers RA, Smith SA. Self-report of hypoglycemia and health-related quality of life in patients with type 1 and type 2 diabetes. Article. *Endocr Pract*. 2013;19(5):792-799. doi:10.4158/ep12382.or
13. Zoungas S, Patel A, Chalmers J, et al. Severe hypoglycemia and risks of vascular events and death. *N Engl J Med*. Oct 7 2010;363(15):1410-8. doi:10.1056/NEJMoa1003795
14. Goto A, Arah OA, Goto M, Terauchi Y, Noda M. Severe hypoglycaemia and cardiovascular disease: systematic review and meta-analysis with bias analysis. Meta-Analysis
Research Support, Non-U.S. Gov't
Review. *BMJ*. 2013;347:f4533. doi:10.1136/bmj.f4533
15. Cryer PE. Death during intensive glycemic therapy of diabetes: mechanisms and implications. Comment
Research Support, N.I.H., Extramural
Research Support, Non-U.S. Gov't. *Am J Med*. Nov 2011;124(11):993-6. doi:10.1016/j.amjmed.2011.08.008

Published under a CC BY license, with permission from Mayo Foundation for Medical Education and Research, original copyright 2025.

16. Khunti K, Davies M, Majeed A, Thorsted BL, Wolden ML, Paul SK. Hypoglycemia and risk of cardiovascular disease and all-cause mortality in insulin-treated people with type 1 and type 2 diabetes: a cohort study. Observational Study
Research Support, Non-U.S. Gov't. *Diabetes Care*. Feb 2015;38(2):316-22. doi:10.2337/dc14-0920
17. Lu C-L, Shen H-N, Hu SC, Wang J-D, Li C-Y. A Population-Based Study of All-Cause Mortality and Cardiovascular Disease in Association With Prior History of Hypoglycemia Among Patients With Type 1 Diabetes. *Diabetes Care*. 2016-07-06 00:00:00 2016;39(9):1571-1578. doi:10.2337/dc15-2418
18. Bonds DE, Miller ME, Bergenstal RM, et al. The association between symptomatic, severe hypoglycaemia and mortality in type 2 diabetes: retrospective epidemiological analysis of the ACCORD study. *BMJ*. 2010;340:b4909. doi:10.1136/bmj.b4909
bmj.b4909 [pii]
19. Seaquist ER, Miller ME, Bonds DE, et al. The impact of frequent and unrecognized hypoglycemia on mortality in the ACCORD study. Research Support, N.I.H., Extramural
Research Support, U.S. Gov't, P.H.S. *Diabetes Care*. Feb 2012;35(2):409-14. doi:10.2337/dc11-0996
20. Patterson CC, Dahlquist G, Harjutsalo V, et al. Early mortality in EURODIAB population-based cohorts of type 1 diabetes diagnosed in childhood since 1989. Multicenter Study
Research Support, Non-U.S. Gov't. *Diabetologia*. Dec 2007;50(12):2439-42. doi:10.1007/s00125-007-0824-8
21. Skrivarhaug T, Bangstad HJ, Stene LC, Sandvik L, Hanssen KF, Joner G. Long-term mortality in a nationwide cohort of childhood-onset type 1 diabetic patients in Norway. Research Support, Non-U.S. Gov't. *Diabetologia*. Feb 2006;49(2):298-305. doi:10.1007/s00125-005-0082-6
22. Gibb FW, Teoh WL, Graham J, Lockman KA. Risk of death following admission to a UK hospital with diabetic ketoacidosis. *Diabetologia*. Jul 11 2016;59(10):2082-2087. doi:10.1007/s00125-016-4034-0
23. Liu S, Zhao Y, Hempe JM, Fonseca V, Shi L. Economic burden of hypoglycemia in patients with Type 2 diabetes. Review. *Expert Rev Pharmacoecon Outcomes Res*. Feb 2012;12(1):47-51. doi:10.1586/erp.11.87
24. Whitmer RA, Karter AJ, Yaffe K, Quesenberry CP, Jr., Selby JV. Hypoglycemic episodes and risk of dementia in older patients with type 2 diabetes mellitus. Research Support, N.I.H., Extramural. *JAMA*. Apr 15 2009;301(15):1565-72. doi:10.1001/jama.2009.460
25. Jacobson AM, Musen G, Ryan CM, et al. Long-term effect of diabetes and its treatment on cognitive function. Randomized Controlled Trial
Research Support, N.I.H., Extramural. *N Engl J Med*. May 3 2007;356(18):1842-52. doi:10.1056/NEJMoa066397
26. Lacy ME, Gilsanz P, Eng C, Beeri MS, Karter AJ, Whitmer RA. Severe Hypoglycemia and Cognitive Function in Older Adults With Type 1 Diabetes: The Study of Longevity in Diabetes (SOLID). *Diabetes Care*. Dec 27 2019;doi:10.2337/dc19-0906
27. Rodriguez-Gutierrez R, Salcido-Montenegro A, Singh-Ospina NM, et al. Documentation of hypoglycemia assessment among adults with diabetes during clinical encounters in primary care and endocrinology practices. *Endocrine*. Dec 4 2019;doi:10.1007/s12020-019-02147-w
28. OptumLabs. *OptumLabs and OptumLabs Data Warehouse (OLDW) Descriptions and Citation. Cambridge, MA: n.p., May 2019. PDF. Reproduced with permission from OptumLabs.* 2019.
29. Wallace PJ, Shah ND, Dennen T, Bleicher PA, Crown WH. Optum Labs: Building A Novel Node In The Learning Health Care System. *Health Aff (Millwood)*. July 1, 2014 2014;33(7):1187-1194. doi:10.1377/hlthaff.2014.0038
30. McCoy RG, Lipska KJ, Yao X, Ross JS, Montori VM, Shah ND. Intensive Treatment and Severe Hypoglycemia Among Adults With Type 2 Diabetes. *JAMA Intern Med*. Jul 1 2016;176(7):969-78. doi:10.1001/jamainternmed.2016.2275

Published under a CC BY license, with permission from Mayo Foundation for Medical Education and Research, original copyright 2025.

31. Pathak RD, Schroeder EB, Seaquist ER, et al. Severe Hypoglycemia Requiring Medical Intervention in a Large Cohort of Adults With Diabetes Receiving Care in U.S. Integrated Health Care Delivery Systems: 2005-2011. *Diabetes Care*. Mar 2016;39(3):363-70. doi:10.2337/dc15-0858
32. Lipska KJ, Ross JS, Wang Y, et al. National trends in US hospital admissions for hyperglycemia and hypoglycemia among Medicare beneficiaries, 1999 to 2011. Journal Article
Research Support, N.I.H., Extramural
Research Support, Non-U.S. Gov't. *JAMA Intern Med*. Jul 2014;174(7):1116-24. doi:http://dx.doi.org/10.1001/jamainternmed.2014.1824
33. Mahoney GK, Henk HJ, McCoy RG. Severe Hypoglycemia Attributable to Intensive Glucose-Lowering Therapy Among US Adults With Diabetes: Population-Based Modeling Study, 2011-2014. *Mayo Clin Proc*. Aug 7 2019;doi:10.1016/j.mayocp.2019.02.028
34. CDC. Diabetes Data & Statistics. Diabetes Atlas. Division of Diabetes Translation, Centers for Disease Control and Prevention, U.S. Dept of Health and Human Services. July 21, 2020. Accessed July 21, 2020. https://gis.cdc.gov/grasp/diabetes/DiabetesAtlas.html#
35. McCoy RG, Lipska KJ, Van Houten HK, Shah ND. Association of Cumulative Multimorbidity, Glycemic Control, and Medication Use With Hypoglycemia-Related Emergency Department Visits and Hospitalizations Among Adults With Diabetes. *JAMA Network Open*. 2020;3(1):e1919099-e1919099. doi:10.1001/jamanetworkopen.2019.19099
36. Sarkar U, Karter AJ, Liu JY, Moffet HH, Adler NE, Schillinger D. Hypoglycemia is more common among type 2 diabetes patients with limited health literacy: the Diabetes Study of Northern California (DISTANCE). *J Gen Intern Med*. Sep 2010;25(9):962-8. doi:10.1007/s11606-010-1389-7
37. Lipska, K J, Warton, et al. HbA1c and risk of severe hypoglycemia in type 2 diabetes: the Diabetes and Aging Study. Research Support, N.I.H., Extramural. *Diabetes Care*. Nov 2013;36(11):3535-42.
38. Karter AJ, Moffet HH, Liu JY, Lipska KJ. Surveillance of hypoglycemia—limitations of emergency department and hospital utilization data. *JAMA Intern Med*. 2018;doi:10.1001/jamainternmed.2018.1014
39. Parsaik AK, Carter RE, Myers LA, Basu A, Kudva YC. Hypoglycemia requiring ambulance services in patients with type 2 diabetes is associated with increased long-term mortality. *Endocr Pract*. Jan-Feb 2013;19(1):29-35. doi:10.4158/ep12197.or
40. Parsaik AK, Carter RE, Myers LA, et al. Population-based study of hypoglycemia in patients with type 1 diabetes mellitus requiring emergency medical services. *Endocr Pract*. Nov-Dec 2012;18(6):834-41. doi:10.4158/ep12094.or
41. Parsaik AK, Carter RE, Myers LA, Dong M, Basu A, Kudva YC. Outcomes of community-dwelling adults without diabetes mellitus who require ambulance services for hypoglycemia. *J Diabetes Sci Technol*. Sep 1 2012;6(5):1107-13. doi:10.1177/193229681200600515
42. Parsaik AK, Carter RE, Pattan V, et al. Population-based study of severe hypoglycemia requiring emergency medical service assistance reveals unique findings. *J Diabetes Sci Technol*. Jan 1 2012;6(1):65-73. doi:10.1177/193229681200600109
43. Ohashi Y, Wolden ML, Hyllested WJ, Brod M. Diabetes management and daily functioning burden of non-severe hypoglycemia in Japanese people treated with insulin. *Journal of Diabetes Investigation*. Feb 08 2017;
44. Brod, M, Rana, A, Barnett, A H. Impact of self-treated hypoglycaemia in type 2 diabetes: a multinational survey in patients and physicians. Clinical Trial Multicenter Study Research Support, Non-U.S. Gov't. *Current Medical Research & Opinion*. Dec 2012;28(12):1947-58.
45. Miller, C D, Phillips, et al. Hypoglycemia in patients with type 2 diabetes mellitus. *Arch Intern Med*. Jul 09 2001;161(13):1653-9.

Published under a CC BY license, with permission from Mayo Foundation for Medical Education and Research, original copyright 2025.

46. McCoy RG, Lipska KJ, Herrin J, Jeffery MM, Krumholz HM, Shah ND. Hospital Readmissions among Commercially Insured and Medicare Advantage Beneficiaries with Diabetes and the Impact of Severe Hypoglycemic and Hyperglycemic Events. *J Gen Intern Med*. Oct 2017;32(10):1097-1105. doi:10.1007/s11606-017-4095-x

47. Karter, A J, Warton, et al. Development and Validation of a Tool to Identify Patients With Type 2 Diabetes at High Risk of Hypoglycemia-Related Emergency Department or Hospital Use. *JAMA Intern Med*. Oct 01 2017;177(10):1461-1470.

48. Festa A, Heller SR, Seaquist E, Duan R, Hadjiyianni I, Fu H. Association between mild and severe hypoglycemia in people with type 2 diabetes initiating insulin. *J Diabetes Complications*. Jun 2017;31(6):1047-1052. doi:10.1016/j.jdiacomp.2016.12.014

49. Quilliam BJ, Simeone JC, Ozbay AB. Risk factors for hypoglycemia-related hospitalization in patients with type 2 diabetes: a nested case-control study. *Clin Ther*. Nov 2011;33(11):1781-91. doi:10.1016/j.clinthera.2011.09.020

50. Davis, T M, Brown, et al. Determinants of severe hypoglycemia complicating type 2 diabetes: the Fremantle diabetes study. Research Support, Non-U.S. Gov't. *Journal of Clinical Endocrinology & Metabolism*. May 2010;95(5):2240-7.

51. Rodriguez-Gutierrez R, Lipska KJ, McCoy RG, et al. Hypoglycemia as an indicator of good diabetes care. *BMJ*. Mar 07 2016;352:i1084. doi:10.1136/bmj.i1084

52. Iezzoni LI, Dorner SC, Ajayi T. Community paramedicine - Addressing questions as programs expand. *N Engl J Med*. 24 Mar 2016;374(12):1107-1109.

53. Rasku T, Kaunonen M, Thyer E, Paavilainen E, Joronen K. The core components of Community Paramedicine - integrated care in primary care setting: a scoping review. Journal Article Review. *Scand J Caring Sci*. Feb 08 2019;08:08. doi:https://dx.doi.org/10.1111/scs.12659

54. Gregg A, Tutek J, Leatherwood MD, et al. Systematic Review of Community Paramedicine and EMS Mobile Integrated Health Care Interventions in the United States. Journal Article. *Popul Health Manag*. Jan 07 2019;07:07. doi:https://dx.doi.org/10.1089/pop.2018.0114

55. Norman GJ, Orton K, Wade A, Morris AM, Slaboda JC. Operation and challenges of home-based medical practices in the US: findings from six aggregated case studies. Journal Article. *BMC Health Serv Res*. 01 27 2018;18(1):45. doi:https://dx.doi.org/10.1186/s12913-018-2855-x

56. Calderone C, Brittain M, Sirivar D, Kotani N. Community Paramedicine Initiative: Transforming Paramedicine in British Columbia. Journal Article. *Stud Health Technol Inform*. 2017;234:54-58.

57. Choi BY, Blumberg C, Williams K. Mobile Integrated Health Care and Community Paramedicine: An Emerging Emergency Medical Services Concept. Journal Article. *Ann Emerg Med*. Mar 2016;67(3):361-6. doi:https://dx.doi.org/10.1016/j.annemergmed.2015.06.005

58. Kizer KW, Shore K, Moulin A. *Community Paramedicine: A Promising Model for Integrating Emergency and Primary Care*. 2013.

59. Guo B, Corabian P, Yan C, Tjosvold L. *Community Paramedicine: Program Characteristics and Evaluation*. Institute of Health Economics; 2017:91. Accessed December 5, 2019. https://www.ncbi.nlm.nih.gov/books/NBK549083/

60. Glenn M, Zoph O, Weidenaar K, et al. State Regulation of Community Paramedicine Programs: A National Analysis. Journal Article. *Prehosp Emerg Care*. Mar-Apr 2018;22(2):244-251. doi:https://dx.doi.org/10.1080/10903127.2017.1371260

61. Glenn M, Zoph O, Weidenaar K, et al. Authority for expanded scope of practice for community paramedics: A national systematic legal review. *Academic Emergency Medicine*. May 2016;1):S76-S77.

62. Bigham BL, Kennedy SM, Drennan I, Morrison LJ. Expanding paramedic scope of practice in the community: a systematic review of the literature. Journal Article

Published under a CC BY license, with permission from Mayo Foundation for Medical Education and Research, original copyright 2025.

Review
Systematic Review. *Prehosp Emerg Care*. Jul-Sep 2013;17(3):361-72. doi:https://dx.doi.org/10.3109/10903127.2013.792890
63. Backstrom C, Ryan J. Community Paramedicine: A Simple Approach To Increasing Access To Care, With Tangible Results. *HealthAffairs* blog. December 5, 2017. https://www.healthaffairs.org/do/10.1377/hblog20171027.424417/full/
64. Wilcox MR. Community Paramedicine in a Rural Setting. Minnesota's approach includes free clinics and a mobile unit that travels the community. Journal Article. *EMS World*. Feb 2016;45(2):17-9.
65. Patterson DG, Coulthard C, Garberson LA, Wingrove G, Larson EH. What Is the Potential of Community Paramedicine to Fill Rural Health Care Gaps? Journal Article. *J Health Care Poor Underserved*. 2016;27(4A):144-158.
66. Stirling CM, O'Meara P, Pedler D, Tourle V, Walker J. Engaging rural communities in health care through a paramedic expanded scope of practice. *Rural and remote health*. 2007 2007;7(4):839.
67. Mi R, Hollander MM, Jones CMC, et al. A randomized controlled trial testing the effectiveness of a paramedic-delivered care transitions intervention to reduce emergency department revisits. Journal Article Randomized Controlled Trial
Research Support, N.I.H., Extramural. *BMC geriatr*. 05 03 2018;18(1):104. doi:https://dx.doi.org/10.1186/s12877-018-0792-5
68. McCarthy P, Brown A, Nystrom P, Ho J. Impact of community paramedic program on health service utilization. *Academic Emergency Medicine*. May 2017;24 (Supplement 1):S112.
69. Huang Y-H, Ma L, Sabljak LA, Puhala ZA. Development of sustainable community paramedicine programmes: a case study in Pennsylvania. Case Reports
Journal Article. *Emerg Med J*. Jun 2018;35(6):372-378. doi:https://dx.doi.org/10.1136/emermed-2017-207211
70. Shah MN, Hollander MM, Jones CM, et al. Improving the ED-to-Home Transition: The Community Paramedic-Delivered Care Transitions Intervention-Preliminary Findings. Journal Article. *J Am Geriatr Soc*. Nov 2018;66(11):2213-2220. doi:https://dx.doi.org/10.1111/jgs.15475
71. Reynolds G, Robinson M, Jernigan M, Webster J, Yehya A. Mobile integrated healthcare - Community paramedicine: An integrated and novel approach to caring for heart failure patients. *Journal of Heart and Lung Transplantation*. April 2018;37 (4 Supplement 1):S314.
72. Snooks HA, Anthony R, Chatters R, et al. Paramedic Assessment of Older Adults After Falls, Including Community Care Referral Pathway: Cluster Randomized Trial. *Ann Emerg Med*. October 2017;70(4):495-505.e28.
73. Kusel E, Savino PB. Boots on the ground. Alameda County, Calif., community paramedics curb hospital readmissions & non-emergent 9-1-1 use. Journal Article. *J Emerg Med Serv JEMS*. Dec 2015;40(12):55-7.
74. Schmitt A, Gahr A, Hermanns N, Kulzer B, Huber J, Haak T. The Diabetes Self-Management Questionnaire (DSMQ): development and evaluation of an instrument to assess diabetes self-care activities associated with glycaemic control. *Health and Quality of Life Outcomes*. 2013/08/13 2013;11(1):138. doi:10.1186/1477-7525-11-138
75. Lorig KR, Sobel DS, Stewart AL, et al. Evidence suggesting that a chronic disease self-management program can improve health status while reducing hospitalization: a randomized trial. *Med Care*. Jan 1999;37(1):5-14. doi:10.1097/00005650-199901000-00003
76. Schmitt A, Reimer A, Hermanns N, et al. Assessing Diabetes Self-Management with the Diabetes Self-Management Questionnaire (DSMQ) Can Help Analyse Behavioural Problems Related to Reduced Glycaemic Control. *PLoS One*. 2016;11(3):e0150774. doi:10.1371/journal.pone.0150774

Published under a CC BY license, with permission from Mayo Foundation for Medical Education and Research, original copyright 2025.

77. Mehravar F, Mansournia MA, Holakouie-Naieni K, Nasli-Esfahani E, Mansournia N, Almasi-Hashiani A. Associations between diabetes self-management and microvascular complications in patients with type 2 diabetes. *Epidemiol Health*. 2016;38:e2016004. doi:10.4178/epih.e2016004 10.4178/epih/e2016004
78. Polonsky WH, Fisher L, Earles J, et al. Assessing psychosocial distress in diabetes: development of the diabetes distress scale. *Diabetes Care*. Mar 2005;28(3):626-31. doi:10.2337/diacare.28.3.626
79. Fisher L, Hessler D, Glasgow RE, et al. REDEEM: a pragmatic trial to reduce diabetes distress. *Diabetes Care*. Sep 2013;36(9):2551-8. doi:10.2337/dc12-2493
80. McEwen LN, Kim C, Haan MN, et al. Are health-related quality-of-life and self-rated health associated with mortality? Insights from Translating Research Into Action for Diabetes (TRIAD). *Primary Care Diabetes*. 2009/02/01/ 2009;3(1):37-42. doi:https://doi.org/10.1016/j.pcd.2009.01.001
81. Clarke PM, Hayes AJ, Glasziou PG, Scott R, Simes J, Keech AC. Using the EQ-5D index score as a predictor of outcomes in patients with type 2 diabetes. *Med Care*. Jan 2009;47(1):61-8. doi:10.1097/MLR.0b013e3181844855
82. Luborsky MR, Rubinstein RL. Sampling in Qualitative Research: Rationale, Issues, and Methods. *Research on aging*. 1995;17(1):89-113. doi:10.1177/0164027595171005
83. Palinkas LA, Horwitz SM, Green CA, Wisdom JP, Duan N, Hoagwood K. Purposeful Sampling for Qualitative Data Collection and Analysis in Mixed Method Implementation Research. *Adm Policy Ment Health*. Sep 2015;42(5):533-44. doi:10.1007/s10488-013-0528-y
84. Guest G, Bunce A, Johnson L. How Many Interviews Are Enough? An Experiment with Data Saturation and Variability. *Field Methods*. February 2006;18(1):59-82. doi:10.1177/1525822X05279903
85. Saunders B, Sim J, Kingstone T, et al. Saturation in qualitative research: exploring its conceptualization and operationalization. *Qual Quant*. 2018;52(4):1893-1907. doi:10.1007/s11135-017-0574-8
86. Sargeant J. Qualitative Research Part II: Participants, Analysis, and Quality Assurance. *Journal of Graduate Medical Education*. 2012;4(1):1-3. doi:10.4300/jgme-d-11-00307.1
87. Hsieh HF, Shannon SE. Three approaches to qualitative content analysis. *Qual Health Res*. Nov 2005;15(9):1277-88. doi:10.1177/1049732305276687
88. Devers KJ. How will we know "good" qualitative research when we see it? Beginning the dialogue in health services research. *Health Serv Res*. Dec 1999;34(5 Pt 2):1153-88.
89. Malterud K. The art and science of clinical knowledge: evidence beyond measures and numbers. *Lancet*. Aug 4 2001;358(9279):397-400.
90. Mays N, Pope C. Qualitative research in health care. Assessing quality in qualitative research. *Bmj*. Jan 1 2000;320(7226):50-2.
91. Patton MQ. *Qualitative Research and Evaluation Methods.* 3rd ed. Sage Publications; 2002.
92. Patton MQ. Enhancing the quality and credibility of qualitative analysis. *Health Serv Res*. 1999;34(5 Pt 2):1189-1208.
93. Braun V, Clarke V. Using thematic analysis in psychology. Article. *Qualitative Research in Psychology*. 2006;3(2):77-101. doi:10.1191/1478088706qp063oa
94. Braun V, Clarke V. What can "thematic analysis" offer health and wellbeing researchers? Editorial. *International Journal of Qualitative Studies on Health and Well-being*. 2014;926152. doi:10.3402/qhw.v9.26152

Published under a CC BY license, with permission from Mayo Foundation for Medical Education and Research, original copyright 2025.
